# High Performance of Photosynthesis and Osmotic Adjustment Are Associated With Salt Tolerance Ability in Rice Carrying Drought Tolerance QTL: Physiological and Co-expression Network Analysis

**DOI:** 10.3389/fpls.2018.01135

**Published:** 2018-08-06

**Authors:** Noppawan Nounjan, Pakkanan Chansongkrow, Varodom Charoensawan, Jonaliza L. Siangliw, Theerayut Toojinda, Supachitra Chadchawan, Piyada Theerakulpisut

**Affiliations:** ^1^Salt-tolerant Rice Research Group, Department of Biology, Faculty of Science, Khon Kaen University, Khon Kaen, Thailand; ^2^Department of Biochemistry, Faculty of Science, Mahidol University, Bangkok, Thailand; ^3^Integrative Computational BioScience Center, Mahidol University, Nakhon Pathom, Thailand; ^4^Rice Gene Discovery Unit, BIOTEC, NSTDA, Kasetsart University, Nakhon Pathom, Thailand; ^5^Plant Biotechnology Research Unit, BIOTEC, NSTDA, Khlong Luang, Thailand; ^6^Center of Excellence in Environment and Plant Physiology, Department of Botany, Faculty of Science, Chulalongkorn University, Bangkok, Thailand

**Keywords:** co-expression network, rice, drought tolerance QTL, osmotic adjustment, photosynthesis, salt stress

## Abstract

Understanding specific biological processes involving in salt tolerance mechanisms is important for improving traits conferring tolerance to salinity, one of the most important abiotic stresses in plants. Under drought and salinity stresses, plants share overlapping responsive mechanisms such as physiological changes and activation of signaling molecules, which induce and transmit signals through regulator genes in a regulatory network. In this study, two near isogenic lines of rice carrying chromosome segments of drought tolerance QTL on chromosome 8 from IR68586-F2-CA-31 (DH103) in the genetic background of sensitive cultivar “Khao Dawk Mali 105; KDML105” (designated as CSSL8-94 and CSSL8-95) were used to investigate physiological responses to salt stress [namely growth, Na^+^/K^+^ ratio, water status, osmotic adjustment, photosynthetic parameters, electrolyte leakage (EL), malondialdehyde (MDA), proline and sugar accumulations], compared with the standard salt tolerant (Pokkali; PK) and their recurrent parent (KDML105) rice cultivars. Physiological examination indicated that both CSSLs showed superior salt-tolerant level to KDML105. Our results suggested that salt tolerance ability of these CSSL lines may be resulted from high performance photosynthesis, better osmotic adjustment, and less oxidative stress damage under salt conditions. Moreover, to explore new candidate genes that might take part in salt tolerance mechanisms, we performed co-expression network analysis for genes identified in the CSSL rice, and found that Os08g419090, the gene involved with tetrapyrrole and porphyrin biosynthetic process (chlorophyll biosynthetic process), Os08g43230 and Os08g43440 (encoded TraB family protein and cytochrome P450, respectively) might have unprecedented roles in salt stress tolerance.

## Introduction

Different types of abiotic stresses pose a great risk to food production and affect crop production worldwide. In Thailand, drought and soil salinity are major causes of low yield of crop cultivation and production, especially in the northeastern part of the country, where rice farming is one of the most important ago-industries, and a major contributing region to the country's rice export. The production of Thai rice, especially Khao Dawk Mali 105 (KDML105 or jasmine rice), one of the most important economic crops in the country, is greatly affected by salinity and water deficit stresses.

Drought and salt in the soil water is known to reduce water uptake ability of plants, causing an osmotic stress (water deficit). Increasing high level of compatible solutes or osmoprotectants (e.g., proline, glycine betaine, and sugar) is believed to adjust the osmotic imbalance when plants are exposed to drought and salinity. Furthermore, water deficit can induce oxidative stress due to overproduction of reactive oxygen species (ROS), which destroy essential molecules and result in membrane damage (Miller et al., 2010). An increase in malondialdehyde (MDA; an indicator for lipid peroxidation) and electrolyte leakage (EL) have been presented in many reports where plants are exposed salinity stress (Sarkar et al., 2013; Mekawy et al., 2015). The specific effect of salt stress on plants is ion toxicity inflicted by over accumulation of Na and Cl ions. The excess of sodium ions disrupts ion transportation in plant cells. Salts enter the transpiration stream and eventually damage cells in the transpiring leaves, further reducing growth (Munns et al., 2006). To survive the detrimental effects of salt stress, plants have evolved many complex mechanisms. The major salt tolerance strategies are (1) osmotic stress tolerance (shoot salt accumulation independent effect) which could prevent effect of salt stress on growth, (2) ion exclusion which contributed to maintaining ion balance, and (3) tissue tolerance which involved Na^+^ compartmentation and high compatible solutes accumulation (Munns and Tester, 2008; Roy et al., 2014).

Two major effects of salt stress on photosynthesis are stomatal closure (the usual response of stomata to salinization of salt-sensitive plants) and a reduction in capacity for CO_2_ fixation as a result of diffusion limitations (Heuer, 2005). When stomata close, CO_2_ levels drop rapidly within the leaf, inhibiting the light-independent reactions. This then causes photosynthesis to stop. Stomatal closure is often a rapid initial response to salt stress (Moradi and Ismail, 2007). Water use efficiency (WUE), defined as the ratio of the rate of carbon assimilation (photosynthesis) to the rate of transpiration, is one of the targets for improvement of crop for enhancing photosynthesis (Flexas et al., 2013). Maximum efficiency at which light absorbed by PSII is used for reduction of primary quinone (Q_A_) electron acceptor of PSII (Fv/Fm) is a parameter used to estimate photosystem II (PSII) photochemistry (Baker, 2008). The reduced Fv/Fm indicated that the photochemical apparatus is damaged under many abiotic stresses (Maxwell and Johnson, 2000).

Thanks to advances in molecular marker breeding, QTLs related to drought tolerance in rice have been identified (Lanceras et al., 2004; Srinivasan et al., 2008). For instance, Kanjoo et al. (2012) have developed improved KDML105 rice lines (CSSL KDML105) with increased drought tolerance but reserved genetic background of KDML105. These chromosome segment substitution lines (CSSL) were introgressed with drought tolerant quantitative trait loci (DT-QTL) on chromosome 8 through a marker assisted backcross breeding between KDML105 and a drought tolerant donor, IR68586-F2-CA-31 line (DH103).

Understanding salt-tolerant level and physiological responses to salt stress of CSSL rice population, which is drought tolerant, may provide new insights that help develop salt and drought tolerant rice in the future. Furthermore, identification of genes involved in salt tolerance mechanism is necessary to gain more knowledge for elucidating gene functions and for designing conventional and biotechnological strategies for improving salt and drought tolerant traits. Therefore, the aim of this work was to investigate salt-tolerant level and physiological responses to salt stress of two CSSL KDML105 DT-QTL8 lines no. 94 and no. 95 (CSSL8-94 and CSSL8-95, respectively) and to identify the potential candidate genes in the DT-QTL segments.

To investigate the functional relationship between genes that potentially have regulatory role in mitigating environmental stresses, co-expression networks have been widely used as a starting point to investigate biological functions of genes in different biological systems including plants. Co-expression network is a graphic representation depicting the coordinated gene expression in response to similar external or internal conditions. Such co-expression patterns of multiple genes across tissues and treatments can be used to predict genetic interactions of related genes. In rice, analysis of co-expressed genes has been used to identify the gene modules that respond to drought and salt stresses (Sircar and Parekh, 2015; Nounjan et al., 2016) and macronutrient deficiency (Takehisa et al., 2015). It can also be used to help characterize the gene functions from the same CSSL lines that might contribute to particular responses observed.

In this study, physiological responses to salt stress of CSSL lines were evaluated by phenotyping salt responsive parameters (growth, Na^+^/K^+^ ratio, water status, osmotic adjustment, photosynthetic parameters, EL, MDA, proline and sugar accumulations) compared to standard salt-tolerant check cultivar, Pokkali (PK) and their recurrent parent, KDML105. PK is well-known for its salt tolerance and has been used for studying physiological, morphological, and biological changes compared to other rice cultivars under salt stress and used as donors of salt-resistance genes for rice breeding (Kanawapee et al., 2012; Sarkar et al., 2013; De Leon et al., 2017). Identification of candidate genes was performed computationally, and their associations with genes of known functions were further characterized using co-expression data of the genes identified in the CSSL lines.

## Materials and methods

### Plant materials and stress treatment

Two lines of CSSL KDML105 DT-QTL8 (CSSL8-94 and CSSL8-95) that received different DT-QTL segments (1 and 2 segments in CSSL8-94 and CSSL8-95, respectively; Supplementary A) were used to analyse co-expression network and study physiological response under salt stress compared to the standard salt-tolerant cultivar, Pokkali (PK; salt-tolerant) and their recurrent parent, KDML105 (salt and drought sensitive). Seeds were germinated in distilled water for 5 days at room temperature, and then transferred to plastic containers containing nutrient solution (Yoshida et al., 1976) which were renewed every 3 days. The pH of nutrient solution was maintained at 5.0–5.5. The experiment was conducted in a greenhouse at the Faculty of Agriculture, Khon Kaen University, Thailand, under natural sunlight conditions. The average day/night temperature was 31°C/19°C, daily mean solar radiation was ~983 μmol photon m^−2^ s^−1^ (PAR), Photoperiod was 11.05 h, and average relative humidity day/night was 42/63%. Salt treatment was started 30 days after germination by replacing with the culture solutions with (salt stress) or without (control) 150 mM NaCl. Electrical conductivity was maintained at 1.70 dS m^−1^ and 16.50 dS m^−1^ in control and salt stress groups, respectively during the entire growth period. Rice plants were sampled after 9 days stress period and analyzed for physiological responses.

### Growth parameters and ion concentration

Dry weight (DW) of whole plants was determined after plants were dried at 70°C for 4–5 days or until the dry weight was stabilized. Dried leaves of the same plant samples (0.25 g) were used for Na and K ions measurement as described by Nounjan et al. (2016). Ion concentration was presented as % on dried weight basis.

### Relative water content, osmotic potential, and osmotic adjustment determination

The relative water content (RWC), osmotic potential (OP), and osmotic adjustment of plants was determined at the mid-day (11.30 a.m.−12.30 p.m.) according to the previous reports (Barrs and Weatherley, 1962; Jongdee et al., 2002). The middle part of a young fully expanded leaf was cut into two pieces (~3 cm long) and immediately weighed for determination of fresh weight (FW). Then, leaf segments were immersed in deionized distilled water in Petri dishes for 12 h at room temperature. Then, leaves were determined for turgid weight (TW) and dried in a hot-air oven at 70°C for 48 h. The dry weight (DW) was determined and RWC was calculated using the equation:

$$\begin{array}{l} \text{RWC}=[(\text{FW}-\text{DW})/(\text{TW}-\text{DW})]\times 100 \end{array}$$

For OP, the same leaf used for determining RWC was used for measuring the osmolality (Jongdee et al., 2002). At harvest the leaf samples were immediately frozen in liquid nitrogen. The collected leaf samples were stored at −20°C for further analysis of osmolality. Leaf sap was expressed from the thawed sample and 10 ml of the leaf sap was used to determine the osmolality (c) using an osmometer (Vapro 5520, Wescor, USA). OP was calculated according to the Van't Hoff equation.

$$\begin{array}{l} \text{OP}=-\text{RTc} \end{array}$$

Where OP is osmotic potential of the leaf sap (MPa), R is the gas constant (0.008341 L MPa mol^−1^ K^−1^) T is 298 K and c is osmolality of leaf sap (mmol/kg H_2_O).

The osmotic adjustment was determined from the difference between OP at full turgor (OP at 100% RWC, OP_100_) of control and stressed plants (Martinez-Ballesta et al., 2004). The OP_100_ was calculated using the following equation described by Turner et al. (1986).

$$\begin{array}{l} {\text{OP}}_{100}=\text{OP}[(\text{RWC}-18)/82] \end{array}$$

### Photosynthetic parameters and chlorophyll content

Net photosynthesis rate (*P*_N_), stomatal conductance (*g*_s_), transpiration rate (*E*) and water use efficiency (WUE) were determined and calculated according to Nounjan et al. (2016). The measurement was performed during 9.30–11:30 at 30°C, 400 ppm of CO_2_ concentration and 37–42% relative humanity using portable gas exchange analyzer (LI-6400 XT, LI-COR, NE, USA). The photosynthetic photon flux density was maintained at 1,500 μmol photons m^−2^ s^−1^. The maximum quantum efficiency of photosystem II (Fv/Fm) was recorded at night (7.00 p.m.) using chlorophyll fluorometer (mini-PAM-II, Walz, Germany). Chlorophyll content was estimated following the method described by Arnon (1949) using 0.1 g of leaf.

### Estimation of EL, MDA, and proline

EL was determined follow the method of Filek et al. (2012). For proline, leaf samples (0.1 g) were used to determine proline concentration according to the method of Bates et al. (1973). MDA was determined by using the method described by Heath and Packer (1968).

### Total soluble sugars determination

The method of Dubey and Singh (1999) was followed to determine total soluble sugars. Briefly, leaf tissues (0.1 g) were extracted in 80% v/v ethanol (three times). One-hundred microliters of supernatant was added to 4 mL of anthrone reagent then incubated at 95°C for 15 min and cooled at room temperature. The absorbance of the green color was measured at 630 nm and the amount of total soluble sugars was calculated by comparison with a standard curve of glucose.

### Gene ontology (GO) enrichment analysis

DT-QTLs in both CSSLs (See Supplementary A) were computationally analyzed to identify the putative genes located in each DT-QTL segment of marker RM447 and RM3480 (Supplementary A) were identified using the Gramene Database (Youens-Clark et al., 2011). To investigate the putative biological functions that the genes within each DT-QTL are involved in, we performed the Gene Ontology (enrichment) analyses using ROAD (Cao et al., 2012) and agriGO (Du et al., 2010) databases, using Rice TIGR gene model as background. Only the GO terms from the biological process category were shown in the results. The enrichments from the two databases were compared using the GO parent-child term relationships from QuickGO database (Binns et al., 2009).

### Identification of putative genes in DT-QTL segments and co-expression network analysis

To explore putative biological processes that the putative genes within the DT-QTL segments are involved in, the genes found in each segment were used as query genes for the co-expression network construction tool in the Rice Oligonucleotide Array Database; ROAD (Cao et al., 2012), under abiotic stress conditions with PCC cutoff of 0.85 and depth of network as a default setting. Next, functional annotations of candidate genes in the DT-QTL segments were investigated using the ROAD database and Rice Genome Annotation Database (Kawahara et al., 2013). Cytoscape (Shannon et al., 2003) was used to visualize and cluster candidate genes in the DT-QTL segments and co-expressed genes, which were colored by their biological processes. Additional co-expression networks were also created using RiceFREND (Sato et al., 2013). Lists of co-expressed genes for each gene were combined into one network with MR cutoff of 30. Genes in the network were annotated and colored according to their biological process GO terms using the GOlorize plugin (Garcia et al., 2007) with only the GO terms with shared parent-child relationships with the terms seen in the initial networks created using the ROAD database, in order to compare them. Parent terms with board definition or at low level were discarded.

### Statistical analysis

For each treatment, the experiment was designed as randomized complete block with four replications. In each container, 12 seedlings per lines/cultivars were randomly arranged. SPSS ver.20 was used for statistical analysis. Data were presented as means ± SD. Significant difference among the treatment means was determined by Duncan's multiple range test (DMRT) (*P* ≤ 0.05). The difference between the means of control and salt stress treatment was determined by Student's *t*-test.

## Results

### Plant growth, ion accumulation, and water status under salt stress

**Figure 1:**
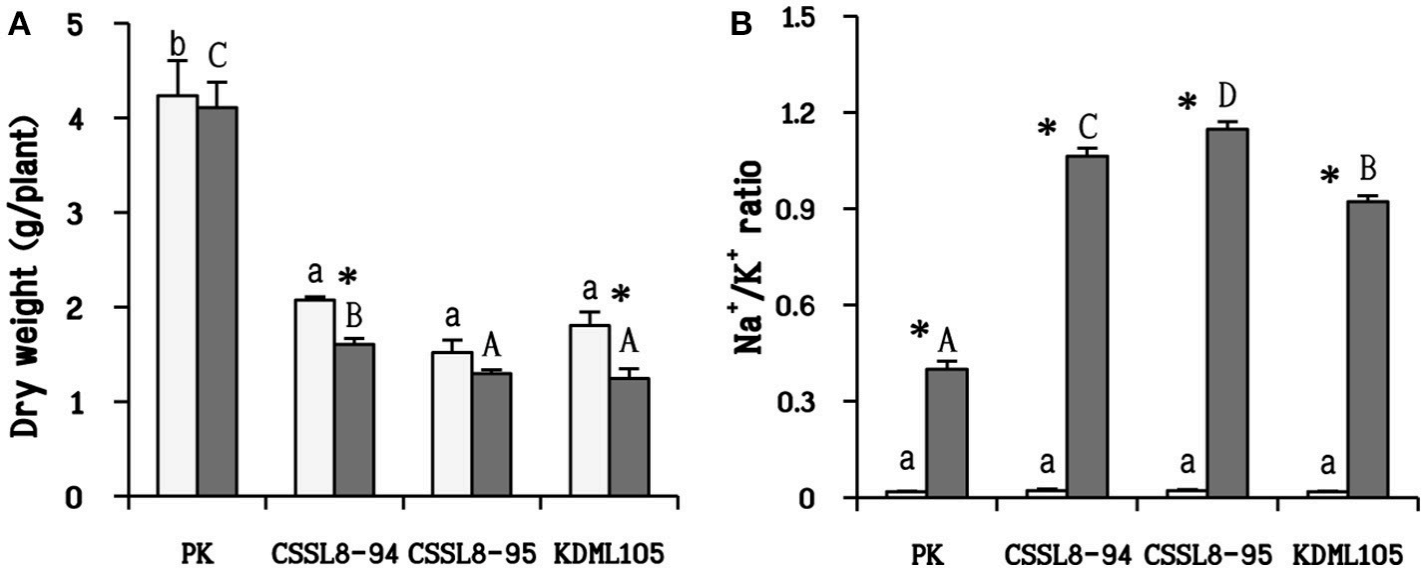
Dry weight **(A)** and Na^+^/K^+^ ratio **(B)** of non-stressed-plants (light bars) and 9 days after salt application (gray bars). The values show means ± SD. For the light bars and gray bars, different lower case and capital letters indicate that the means are significantly different (*P* ≤ 0.05). The significant difference (*P* ≤ 0.05) of values between control and stressed plants is indicated by asterisk (*).

**Figure 2:**
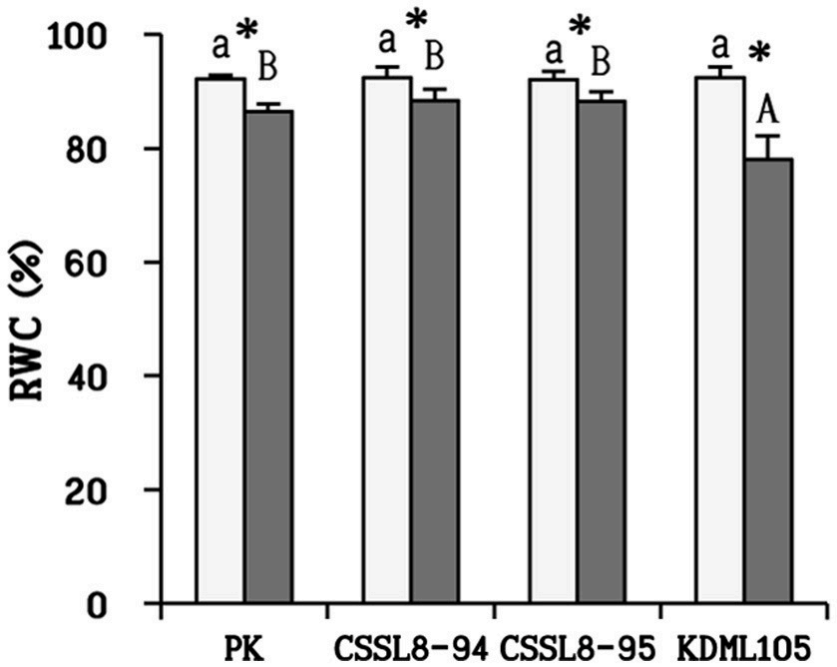
Relative water content (RWC) in plants at normal conditions (light bars) and 9 days after salt stress (gray bars). The values show means ± SD. For details of statistical symbols, see Figure 1.

There was no significant reduction in dry weights of salt-stressed PK and CSSL8-95 compared to those of the control groups while a significant decline in dry weight was observed CSSL8-94 and KDML105. The most prominent decrease in dry weight was found in KDML105 (decreased by 31% compared to control). Salt-stressed CSSL8-94 showed higher dry weight than CSSL8-95 and KDML105, even though the percentage of reduction was higher than that of the salt-stressed CSSL8-95 (Figure 1A). The Na^+^/K^+^ ratios were not different in all rice lines/cultivars under the control condition but markedly increased under salt stress; 57.5, 53, 46, and 20-folds increase in CSSL8-95, CSSL8-94, KDML105, and PK, respectively (Figure 1B). Salt treatment resulted in significant reductions in RWC in all rice lines/cultivars with the most pronounced decrease in KDML105 (Figure 2).

### Photosynthetic parameters and chlorophyll under salt stress

**Figure 3:**
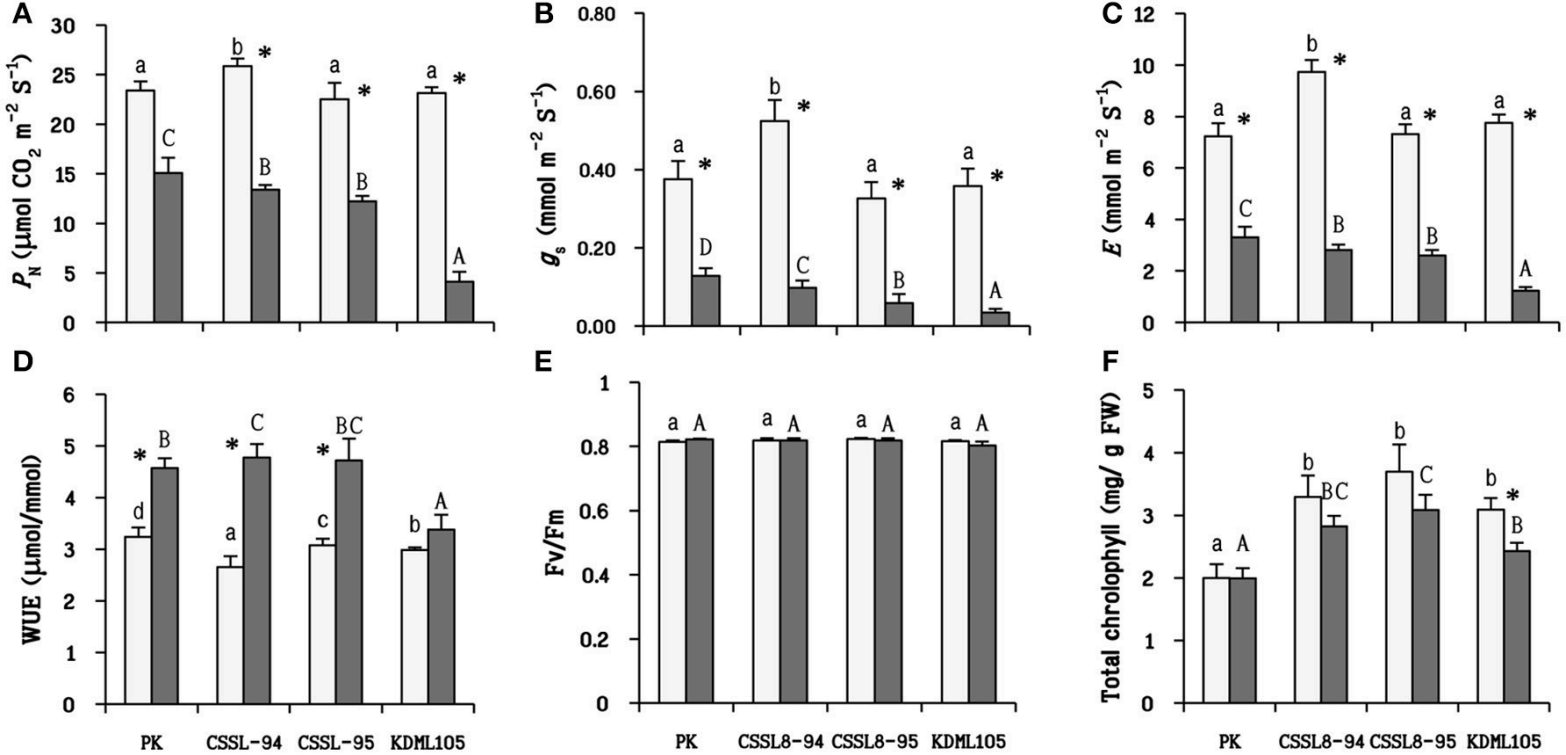
Photosynthetic parameters in plants net photosynthesis rate **(A)** stomatal conductance **(B)** transpiration rate **(C)** water use efficiency **(D)** the maximum quantum yield of PSII **(E)** and total chlorophyll **(F)** at normal conditions (light bars) and 9 days after salt stress (gray bars). The values show means ± SD. For details of statistical symbols, see Figure 1.

Under control condition, CSSL8-94 exhibited the highest photosynthetic parameters (*P*_N_, *g*_s_, and *E*) compared to other rice lines/cultivars. When plants were challenged with salt stress, it can be clearly seen that salt stress resulted in a significant decline in photosynthetic parameters in all rice lines/cultivars. PK showed the highest photosynthetic parameters, followed by the CSSL lines and KDML105 in salt treatment conditions (Figures 3A–C). Conversely, WUE increased markedly under the salt treatment. The level of WUE in CSSL8-94 increased about 45% after salinity stress, 35% for CSSL8-95, and 29% in PK. WUE of salt-stressed KDML105 increased by only 12% compared with the control (Figure 3D). For Fv/Fm, no significant differences were observed between untreated plants and stressed plants (Figure 3E). Chlorophyll content of PK remained unchanged when plants were treated with NaCl compared to untreated plants. Decreased chlorophyll content was noted in both CSSL8 lines but was not significantly different compared to those of control groups while that in KDML105 reduced markedly from controlled plants (21% decreased) (Figure 3F).

### Osmotic adjustment and osmolytes (proline and total sugar) under salt stress

**Figure 4:**
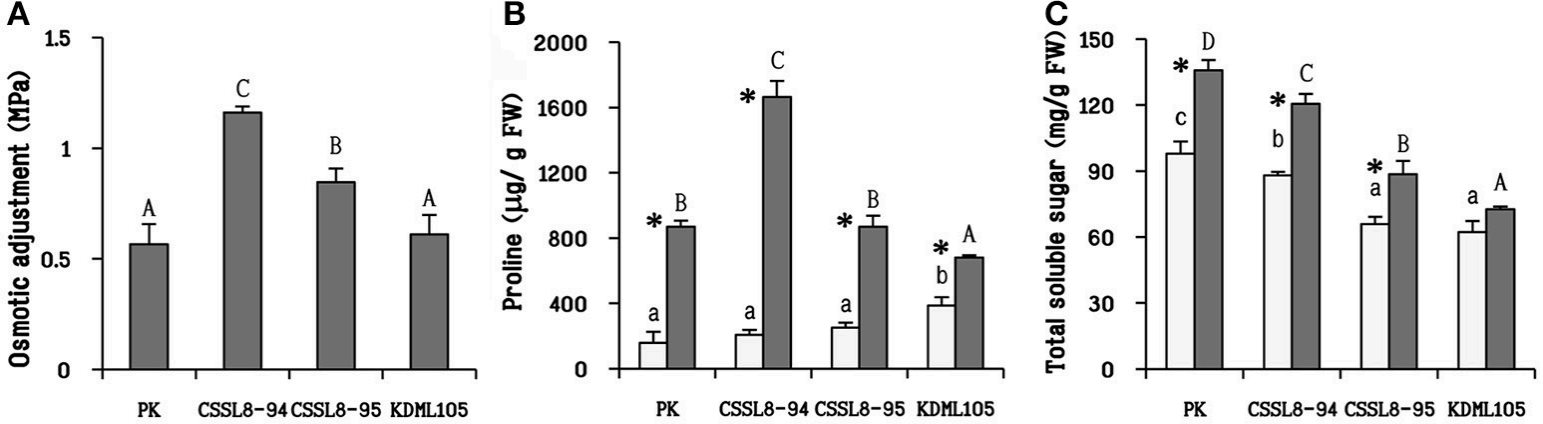
Osmotic adjustment **(A)** and osmolytes (proline and total soluble sugar; **B,C**, respectively) at normal condition (light bars) and 9 days after salt stress (gray bars). The values show means ± SD. For details of statistical symbols, see Figure 1.

The highest osmotic adjustment level was observed in CSSL8-94 (1.16 MPa) followed by CSSL8-95 (0.85 MPa). Similar level of osmotic adjustment, significantly lower than those of the CSSLs, was observed for PK and KDML105 (Figure 4A). A drastic increase in proline accumulation was found in all stressed plants. The increments in proline were maximum in CSSL8-94 (8.4 times) followed by PK (5.5 times), CSSL8-95 (3.5 times), and then KDML105 (1.8 times only) (Figure 4B). Accumulation of total sugar was also observed in all rice lines/cultivars under salt stress condition. The amounts of total sugar were significantly increased, compared to untreated plants, in PK, CSSL8-94, and CSSL8-95 (28, 27, and 26%, respectively) while a slight increase (14%) was noted in KDML105 (Figure 4C).

### EL, MDA content under salt stress

**Figure 5:**
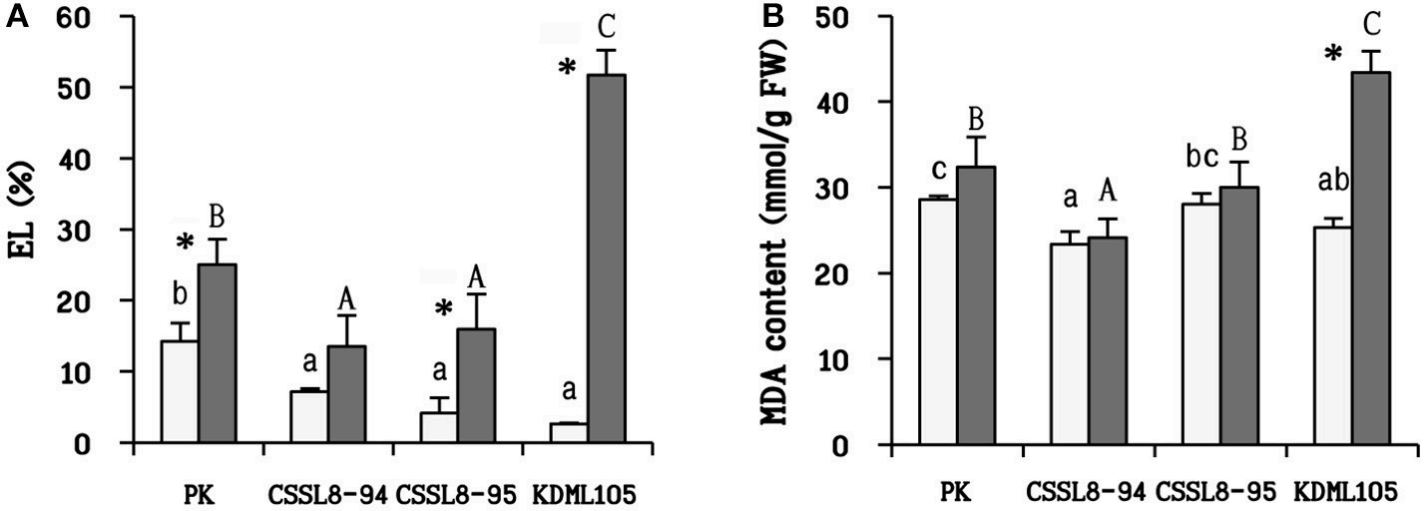
Electrolyte leakage (EL) **(A)** and malondialdehyde (MDA) content **(B)** at normal condition (light bars) and 9 days after salt stress (gray bars). The values show means ± SD. For details of statistical symbols, see Figure 1.

After salt treatment, the percentage of EL was observed to be increased in all rice lines/cultivars (increased 43% in PK, 47% in CSSL8-94, 74% in CSSL8-95, and 95% in KDML105) (Figure 5A). MDA content in salt-stressed KDML105 increased significantly (increased 42% compared to control) and accumulated to a much higher concentration than in other rice lines/cultivars. In contrast, MDA content was slightly increased in PK, CSSL8-94, and CSSL8-95 (Figure 5B).

### Identification of putative genes in DT-QTL segments

**Figure 6:**
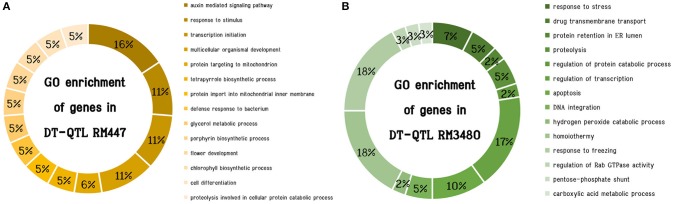
Significantly enriched GO enrichment (*P* < 0.05) of genes in DT-QTL. The chart shows distribution based on biological process of genes in RM447 **(A)** and RM3470 **(B)**.

To investigate the genomic regions that might influence drought-tolerant traits in the CSSL lines, we first searched for the genes within each DT-QTL segment using the Gramene Database (Youens-Clark et al., 2011). There are in total of 79 and 78 putative genes in RM447 and RM3480, respectively (Supplementary B). We then explored possible mechanisms that these genes might be related to salt tolerance by Gene Ontology (GO) terms enrichment analysis. For those with multiple GO terms, the term that was also found most frequently among other genes in the segment was chosen to represent that gene in Figure 6. Based on GO term enrichment analysis performed using ROAD (Cao et al., 2012) (*P* < 0.05), 14 significant functional groups were observed in each DT-QTL. There are certain differences in the enrichment of biological processes in both DT-QTL regions. For example, the major GO terms representing putative genes found in RM447 are auxin mediated signaling pathway, followed by response to stimulus, transcription initiation and multicellular organismal development (Figure 6A) (Supplementary C). For RM3480, the major significantly enriched GO terms are divided into three categories: homoiothermy, response to freezing, and regulation of transcription (Figure 6B). In addition, we also performed the GO enrichment analyses using an alternative data, agriGO (Du et al., 2010) for RM447 and RM3480 (Supplementary E), as well as for random genes (Supplementary F). Interestingly, we found that the GO terms from both sources for the RM447 and RM348 segments are highly concordant, or at least closely linked based on GO parent-child relationships, whereas the same is not true when randomly picked genes were used (Supplementary G, H). These together suggest that the GO term enrichment analysis performed was robust, and that the enriched terms might be useful to further investigate the pathways involving salt tolerance in these CSSLs.

### Co-expression network of putative genes in DT-QTL associated with abiotic stress

**Figure 7:**
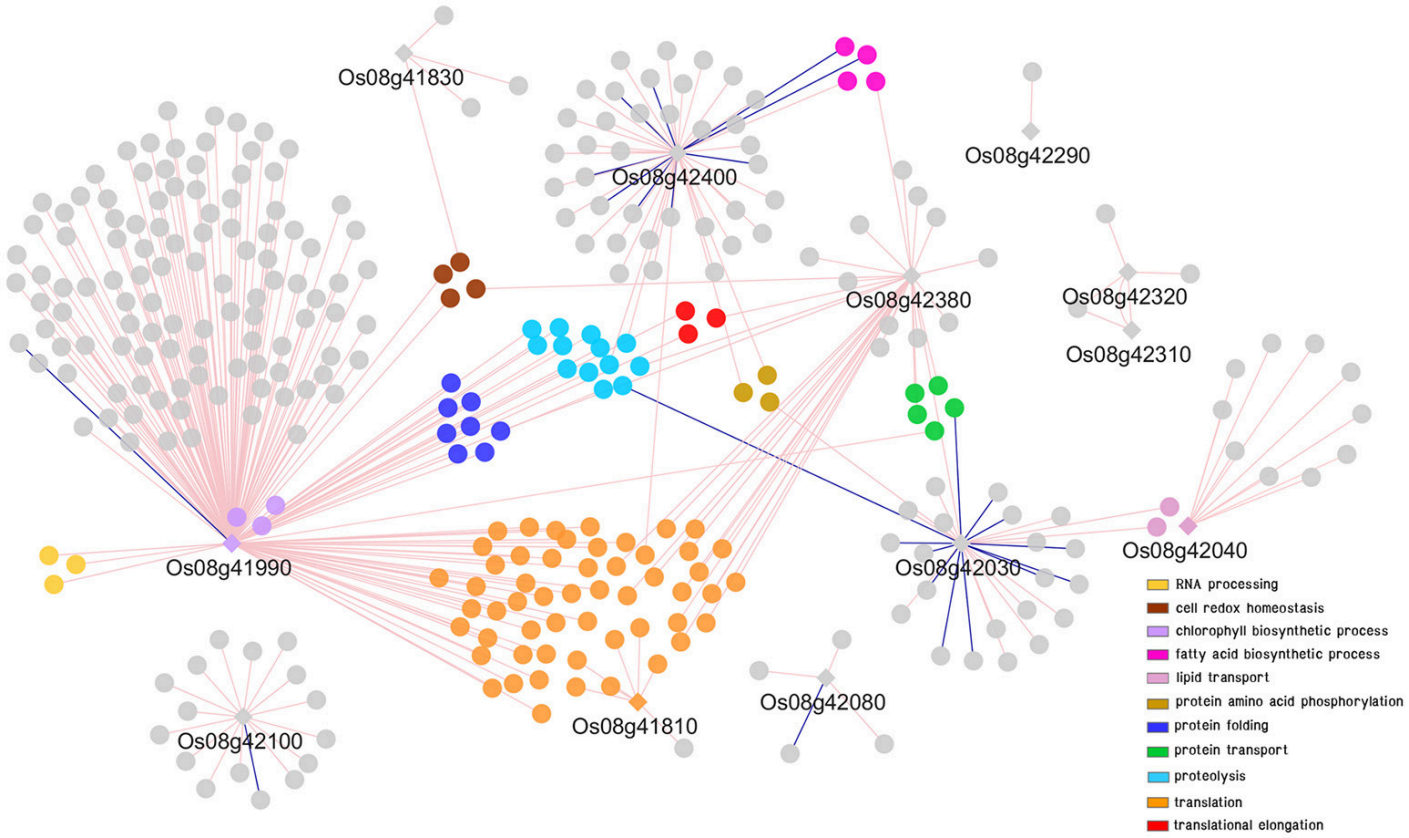
Co-expression network analysis of genes in RM447 under abiotic stress condition. Round shapes represent genes (node); diamond shape represents the putative genes found in DT-QTL. Pale pink line (edge) links two genes that are positively correlated. Dark blue line (edge) links two genes that are negatively correlated. GO term enrichment of genes is represented in different colors.

**Figure 8:**
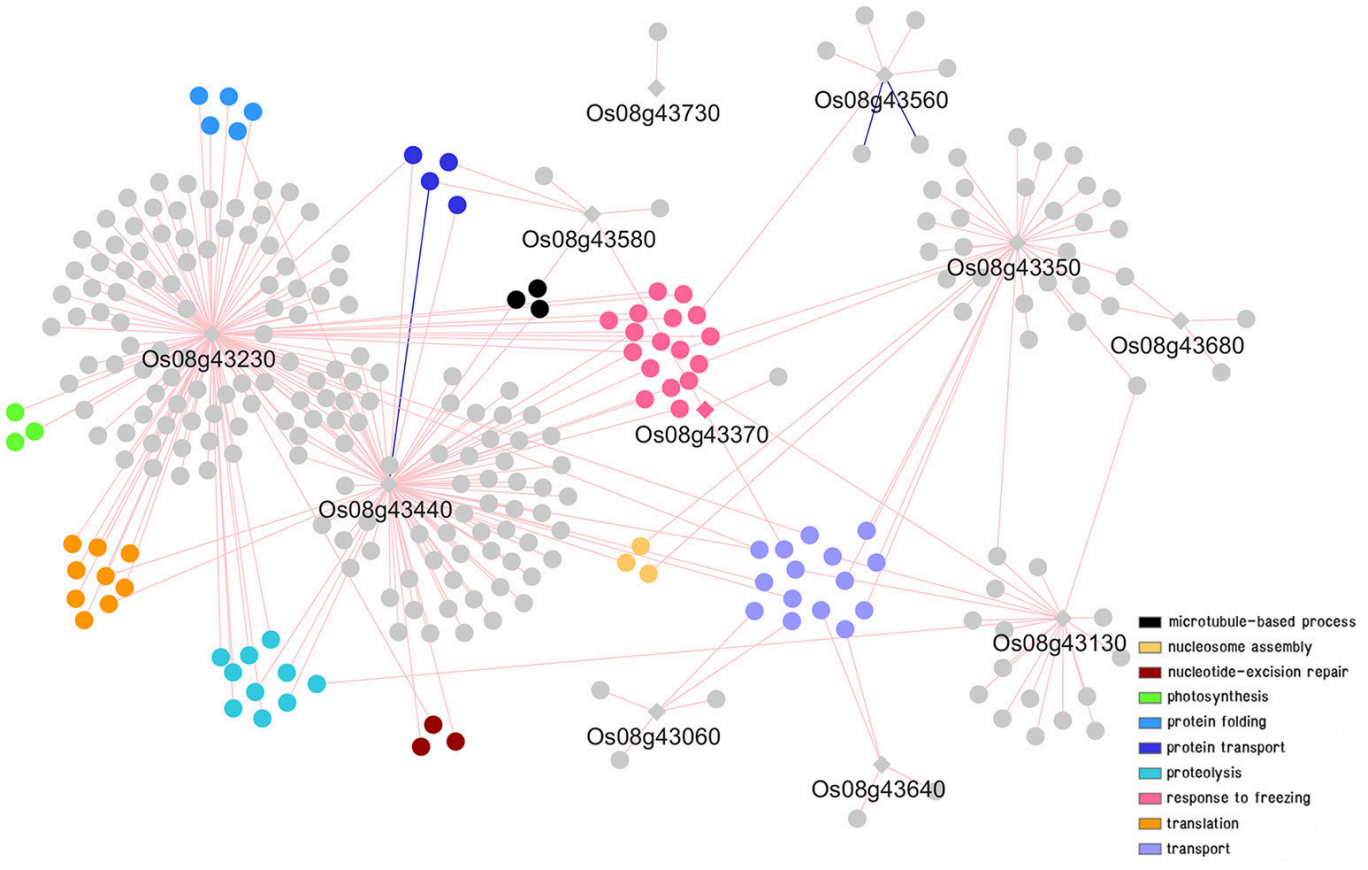
Co-expression network analysis of genes in RM3480 under abiotic stress conditions. For details of symbols and colors, see Figure 7.

To further explored possible functions and pathways that the putative genes identified might be involved in, co-expression networks of genes in DT-QTL were constructed from the putative genes found in RM447 and RM3480, and other genes that showed similar expression patterns under abiotic stresses (see section Materials and Methods). Our rice abiotic stress co-expression networks of RM477 contains 12 putative genes in the segment whose expression patterns are co-expressed with 305 genes (nodes); whereas the 11 putative genes from RM3480 have 288 co-expressed genes (Figures 7, 8). The list of genes in DT-QTL that are significantly co-expressed with other genes is shown in Table 1. Additional co-expression networks were also constructed using an alternative database, RiceFREND (Sato et al., 2013). The networks and comparison between two databases of the RM447 and RM3480 segments, as well as randomly picked genes, can be found in Supplementary Materials (Supplementary I–P).

**Table 1:** List of genes in DT-QTL (RM447 and RM3480) co-expressed with other genes in rice under abiotic stress condition (PCC 0.85).

**QTL location**	**Locus ID**	**RGAP Ver 6 Annotation**	**GO name (GO level)**	**No. of genes in module**	
				**Positive correlation**	**Negative correlation**
RM447	Os08g41810	Ribosomal protein L22	Translation (5,6)	7	–
	Os08g41830	Sulfite oxidase	Oxidation reduction (3)	4	–
	Os08g41990	Aminotransferase	Porphyrin biosynthetic process (6), chlorophyll biosynthetic process (5), tetrapyrrole biosynthetic process (5)	168	1
	Os08g42030	Peroxidase precursor	Response to oxidative stress (4)	15	12
	Os08g42040	LTPL80 - Protease inhibitor/seed storage/LTP family protein precursor	Lipid transport (5)	10	–
	Os08g42080	ACR5	Metabolic process (2)	3	1
	Os08g42100	ACT domain containing protein	Metabolic process (2)	15	1
	Os08g42290	expressed protein	No GO terms found	1	–
	Os08g42310	Expressed protein	No GO terms found	2	–
	Os08g42320	Expressed protein	No GO terms found	4	–
	Os08g42380	Mitochondrial import inner membrane translocase subunit Tim	Protein targeting to mitochondrion (6,7), protein import into mitochondrial inner membrane (7,9)	32	–
	Os08g42400	No apical meristem protein	Regulation of transcription (–)	36	9
RM3480	Os08g43060	Expressed protein	Carboxylic acid metabolic process (6)	5	–
	Os08g43130	NAP1	No GO terms found	20	–
	Os08g43230	TraB family protein	No GO terms found	128	–
	Os08g43350	NC domain-containing protein	No GO terms found	37	
	Os08g43370	6-phosphogluconolactonase	Pentose-phosphate shunt (5)	1	–
	Os08g43440	Cytochrome P450	Oxidation reduction (3)	91	1
	Os08g43560	OsAPx4 - Peroxisomal Ascorbate Peroxidase encoding gene 5,8,9	Response to oxidative stress (4), hydrogen peroxide catabolic process (6)	5	2
	Os08g43580	Acyl carrier protein	Fatty acid biosynthetic process (6), lipid biosynthetic process (4)	6	–
	Os08g43640	26S proteasome non-ATPase regulatory subunit 3	Regulation of protein catabolic process (6)	4	–
	Os08g43680	Glutathione S-transferase, C-terminal domain containing protein	No GO terms found	4	–
	Os08g43730	DUF630/DUF632 domains containing protein	No GO terms found	1	–

#### Co-expression network of putative genes in Rm447

The GO term enrichment analysis of gene co-expression relationships (*P* < 0.05) with the putative genes identified in RM447 reveals that its predominant biological functions are translation (orange) followed by proteolysis (light blue), protein folding (dark blue), and protein transport (green), respectively (Figure 7) (Supplementary D, I, M).

The putative genes in RM447 with the highest numbers of co-expressed genes based on the ROAD database were Os08g41990 (aminotransferase), Os08g42380 (mitochondrial import inner membrane translocase subunit Tim), and Os08g42400 (no apical meristem protein) (Figure 7; Table 1). Functional analysis shows that Os08g41990 itself is related to chlorophyll biosynthetic process, porphyrin, and tetrapyrrole biosynthetic process. The gene shows similar co-expression pattern with 169 genes (168 genes are found to be positively correlated while one gene is negatively correlated with Os08g41990). Other genes with positive co-expression pattern with Os08g41990 are chloroplast precursor proteins, and RNA processing. For example, a chloroplast precursor protein, Os02g07230, is predicted to have similar functions as Os08g41990. Others genes co-expressed with Os08g41990 are involved in proteolysis, including Clp protease group (Os01g16530, Os01g32350, Os03g22430, and Os10g43050), M24 family protein (Os04g52100, Os07g32590), and Deg protease protein (Os05g05480 and Os12g42210). Note that the number of co-expressed genes was significantly lower when RiceFREND was used instead of ROAD; however, both databases suggested that Os08g41990 had similar expression patterns to genes involving in RNA processing (shown in yellow; Supplementary I).

For Os08g42380, it shows similar expression pattern with other 32 genes based on ROAD (Figure 7; Table 1). This gene is annotated as mitochondrial import inner membrane translocase subunit Tim and may function in mitochondria (cellular component result shows that this gene located in mitochondrial intermembrane space protein transporter complex; Cao et al., 2012). The genes co-expressed with Os08g42380 can be divided into two main groups based on GO enrichment analysis; translation (i.e., Os01g14830 and Os02g56014) and protein transport (Os02g45820, Os03g61019, and Os12g38310). Co-expression network from RiceFREND confirmed the link between Os08g42380 and co-expressed genes involving in translation.

For Os08g42400 (no apical meristem), this gene is annotated as being involved in regulation of transcription but its location has not yet been identified. Our co-expression analysis showed that 36 and 9 genes are positively and negatively correlated with Os08g42400 (Figure 7; Table 1). Our GO term analysis of co-expressed genes with Os08g42400 showed the group of genes involving regulation of transcription (such as homeobox domain containing protein; Os03g03260, Os03g03260, Os06g43860, and Os10g01470), fatty acid biosynthetic process (Os02g51150, Os04g55060, and Os07g01150) and iron-sulfur cluster assembly (Os01g47340).

The co-expression network of genes constructed from genes found in RM447 also shows some genes involved in response to oxidative stress, such as Os08g42030 (peroxidase precursor). Os08g42030 is positively and negatively co-expressed with 15 and 12 genes, respectively (Figure 7; Table 1). The most enriched GO terms of genes co-expressed with Os08g42030 is involved in lipid transport (Os10g40430 and Os10g40614). Such patterns were not present when randomly selected genes were used to build the co-expression network (Supplementary J, O). Overall, our network representation depicts the dominance of co-expressed genes involving translation, proteolysis, and protein folding, which might be linked to the salt stress property in CSSL8-95.

#### Co-expression network of putative genes in RM3480

In the case of RM3480, GO term enrichments were found to be abundant in four main categories: response to freezing (pink), transport (purple), proteolysis (light blue), and translation (orange) (Figure 8; Supplementary D, K, N). A gene in RM3480, Os08g43230 (TraB family protein) is positively co-expressed with 128 genes in the network under abiotic stresses based on the ROAD database (Figure 8; Table 1). Those genes function in photosynthesis (Os01g59090, Os03g52130, and Os06g04150), proteolysis (i.e., Os01g47450, Os04g52100, and Os12g42210), response to freezing and homoiothermy (i.e., Os02g07350, Os06g02580, and Os08g25700).

For Os08g43440, which encodes cytochrome P450 associated with oxidation reduction shows, positive and negative expression with 91 genes and 1 gene, respectively based on ROAD (Figure 8; Table 1). The expression of Os08g43440 itself also shows similar expression pattern to another candidate gene in the segment, Os08g43230. The expression similarity between two genes within RM3480; however, was not observed in the network made using RiceFREND, which contains significantly fewer interactions. Os08g43440 is linked with genes functioning in oxidation reduction (i.e., Os08g44810 and Os09g39390), hydrogen peroxide catabolic process related genes (Os02g34810and Os03g03910) and iron-sulfur cluster assembly gene (Os03g20010 and Os06g47940).

For Os08g43350 (NC domain-containing protein), this gene is positively correlated with 37 genes (Figure 8; Table 1), which GO enrichment analysis revealed their functions in nucleosome assembly (Os02g45940, Os05g36280, and Os07g40130) and microtubule-based movement (Os02g53520 and Os10g36880).

Other co-expression analyses also showed that Os08g43560 (OsAPx4 –peroxisomal ascorbate peroxidase encoding gene 5, 8, 9) is positively and negatively correlated with 5 and 2 genes, respectively (Figure 8; Table 1). Os08g43560 plays a role in response to oxidative stress and hydrogen peroxide catabolic process linked with genes involved in iron-sulfur cluster assembly (Os06g05400). Such patterns were not present when randomly selected genes were used to build the co-expression network (Supplementary L, P). Overall, we found that the most abundant biological functions of genes that show similar expression patterns under abiotic stresses to the putative genes in the segment RM3480 are response mainly to freezing, transport, proteolysis, and translation.

## Discussion

### CSSL rice showed better growth than their recurrent parent, KDML105, despite less efficient ion exclusion

Salt stress is known to decrease water uptake ability in plants leading to reduced plant growth and accumulation of toxic ions resulting in impaired metabolic processes. In this study, salt stress had no effects on dry weight of PK and CSSL8-95 but significantly reduced dry weights of KDML105 (31%) and CSSL8-94 (23%) (Figure 1A). This study corroborated previous results on studying physiological responses to salt stress of selected CSSL8 lines compared with their parental lines (DT-QTL donor; DH103 and recurrent parent; KDML105) with the finding that CSSL8-94 showed less salt stress damage and growth reduction than KDML105 (Nounjan et al., 2016). In the case of CSSL8-95, the result showed that dry weight of CSSL8-95 was not significantly reduced compared to untreated plants. This observation indicated that CSSL8-95 had better tolerance than KDML105 (Figure 1A).

Both CSSL lines showed higher Na^+^/K^+^ ratio than PK and KDML105 (Figure 1B). The lowest Na^+^/K^+^ ratio in PK was due to lower Na^+^ and higher K^+^ accumulation (Figure 1B). This cultivar might prevent negative effect from NaCl by ion exclusion that is one of the most important salt tolerance mechanisms in plants to reduce Na^+^ and Cl^−^ uptake (Roy et al., 2014). Numerous studies have reported that salt-tolerant rice genotypes, particularly PK, had lower Na^+^/K^+^ ratio than salt susceptible ones (Kanawapee et al., 2012). This difference was also clearly observed in this present study between PK and other rice lines/cultivars. Although the Na^+^/K^+^ ratios observed in CSSL8-94 and CSSL8-95 were higher than that of KDML105, they showed better growth than KDML105 (Figures 1A,B). In fact, salt tolerance mechanism in plants consists of many mechanisms such as ion exclusion, osmotic, and tissue tolerance (Roy et al., 2014). This observation might be resulted from tissue tolerance mechanism which could alleviate plant cells from ion toxicity by Na ion compartmentation. Also, Pires et al. (2015) has pointed out that Na ion content was not the only key to distinguish salt tolerance level in plants. Genotypes with similar levels of Na accumulation in leaves showed high variability in physiological response and tolerance level.

In addition, synthesis of compatible solutes (such as proline) has been reported to involve in tissue tolerance mechanisms in plants (Munns and Tester, 2008; Roy et al., 2014). Under salt treatment, CSSL8-94 had highest proline content, in contrast to KDML105 while proline content of CSSL8-95 and PK were in between (Figure 4B). Many studies have demonstrated that higher proline content was noted in salt-tolerant than salt-sensitive genotypes (Kumar et al., 2010; Mansour and Ali, 2017). High proline content observed in both CSSL8 lines might promote better growth compared to recurrent parent, KDML105 though they showed higher Na^+^/K^+^ ratio (Figures 1, 4B). This finding indicates that both CSSLs tested here appeared to have better salt tolerance level than their recurrent parent, KDML105. Nevertheless, ion exclusion process may not be the major contributor to salt tolerance mechanism in CSSL lines.

### Maintenance of water status and good osmotic adjustment contributed to salt tolerance ability in CSSL rice

Water is undoubtedly the most important factor determining normal plant metabolic, biochemical, cellular, and whole-plant functions. Salinity stress disturbs the balance of water status in plants. RWC can be used to describe the water status in plant cells (Negrão et al., 2017). Maximum decrease in RWC was found in KDML105 compared to other rice plants (Figure 2). Many studies have reported that tolerant genotypes had less decrease in RWC than sensitive ones (Hossain et al., 2015; Mekawy et al., 2015). Therefore, this finding indicated that KDML105 had less ability in maintenance of water status which subsequently affected other physiological responses (such as the ability to maintain photosynthetic process, increase WUE and osmotic adjustment; Figures 3, 4A) leading to decrease the ability to resist salt stress.

### Well-maintained photosynthesis process and photosynthetic pigment associated to salt tolerance mechanism in CSSL rice

Salt-stressed PK was found to attain the highest *P*_N_ and have lowest reduction percentage compared to the controls, and KDML105 was the most severely affected. The *P*_N_ of salt-stressed CSSL8-94 and CSSL8-95 were intermediate between the salt-tolerant, PK and the salt-sensitive, KDML105 (Figures 3A–C). The ability of PK to efficiently maintain photosynthesis capacity was previously observed (Boriboonkaset et al., 2013; Sarkar et al., 2013). Huang et al. (2014) and Ma et al. (2016) also reported that salt-tolerant genotypes had a higher *P*_N_ than salt-sensitive one under salt and drought stress in ramie grass and rice, respectively.

For WUE, it was found that WUE increased under salt stress. It has been reported that tolerant genotypes had higher WUE than sensitive genotypes under stressful environments (Chandrakala et al., 2013; Flexas et al., 2013). In agreement with those reports, WUE in PK and both CSSL8 lines were significantly higher than that in KDML105 under salt stress (Figure 3D).

Decreased Fv/Fm indicates that PSII reaction center is damaged subsequent to photoinhibition (Murata et al., 2007). In this study, Fv/Fm was unaffected when plants were supplemented with NaCl (Figure 3E). Thus, this result indicated that PSII photochemical apparatus in plants were not injured bysalt stress.

Reduction of total chlorophyll occurred in all salt-stressed plants, except for PK. However, only the KDML105 cultivar displayed significant decrease in total chlorophyll (Figure 3F). This observation was consistent with many reports that indicated that salt stress decreased chlorophyll content in plants (Khan et al., 2015; Nounjan et al., 2016). Evaluation of salt tolerance in rice genotypes using physiological characters including chlorophyll content showed that salt-tolerant and moderately tolerant genotypes contained significantly higher chlorophyll content than moderately sensitive, sensitive and highly sensitive genotypes under saline conditions (Kanawapee et al., 2012). Therefore, the results obtained in this work suggested that salt stress induced less damage in both CSSL8 lines than in KDML105.

With respect to the observed photosynthetic parameters, it is considered that CSSL8-94 and CSSL8-95 had better salt-tolerant level than that of the parental cultivar KDML105. The previous report using soil-grown plants at the young seedling stage on the same CSSL population by Nounjan et al. (2016) has pointed out that the DT-QTL donor (DH103) and the salt-tolerant CSSL line (CSSL8-94) exhibited higher *P*_N_, *g*_*s*_, *E*, and WUE than salt sensitive CSSL line (CSSL8-116) under saline condition. The ultimate aim of improving crop abiotic stress tolerance is to obtain higher yield. Photosynthetic capacity is directly linked to yield (Ambavaram et al., 2014). A recent experiment study on yield components under salt stress using subset of this CSSL KDML105 DT-QTL8 population found thatCSSL8-94had better fertile spikelet, seed setting, grain yield and harvest index than its recurrent parent KDML105 (unpublished data).

### High concentration of osmolytes (proline and total sugar) and less membrane damage contributed salt tolerance ability in CSSL rice

Osmotic adjustment is one of the mechanisms for conserving plant cells hydration under drought and salinity stresses. Occurrence of high proline content, especially in both CSSL8 lines treated with NaCl could associate with high osmotic adjustment observation in contrast to KDML105 which accumulated less proline and exhibited low osmotic adjustment (Figures 4A,B). It is known that high accumulation of compatible solute such as proline under salt stress involved in adaptation to tolerate osmotic effects leading to reduce the cellular water potential to a level below the external water potential (Hossain et al., 2014).

Moreover, proline has also been reported for its beneficial roles such as ROS scavenging molecule to protect plants under abiotic stress (Hossain et al., 2014). High proline content in PK and both CSSL8 lines could be alleviating the adverse effect of oxidative damage in plant cells leading to the observation of lower EL and a slight increase in MDA content in those plants compared to KDML105 when plants were exposed to salt stress (Figures 5A,B). Liang et al. (2013) summarized that proline can react with H_2_O_2_ and OH^•^ to form stable free radical adducts of proline and hydroxyproline derivatives. These observations indicated that salt stress has more negative impact on KDML105 than both CSSL8 lines and proline plays beneficial roles to mitigate ROS-related effects of salt stress on both CSSL8 lines. Therefore, it is suggested that proline accumulation involved in osmoprotectant and ROS scavenger functions.

Significant increase in total soluble sugar was found in all rice lines/cultivars under salt stress except for KDML105 (Figure 4C). Higher total soluble sugars have been reported in salt-tolerant genotypes than salt-sensitive ones (Nemati et al., 2011; Khan et al., 2015). Sugar can act as an osmolyte to preserve membrane integrity and act as a signaling molecule to induce metabolic rearrangements and regulatory networks under stressful environmental stress (Krasensky and Jonak, 2012). Higher total soluble sugar in salt-stressed CSSL8-94 and CSSL8-95 compared to those in KDML105 was associated with higher osmotic adjustment in those lines. In accordance with those results, it was suggested that sugar might play a role in osmotic adjustment mechanism in both CSSL8 lines under salinity stress resulting in better salt tolerance than KDML105 (Figures 4A,C). Moreover, Lee et al. (2013) presented that proline and sucrose were higher accumulated in plants grown under osmotic stress, and higher concentrations of compatible solutes are related to the severity of osmotic stress indicating the presence of osmotic adjustment mechanism.

ROS leads to cellular membrane damage (indicated by EL) and lipid peroxidation (indicated by MDA content). In this present study, increasing in EL was found in all rice lines/cultivars (especially in KDML105) except for CSSL8-94. PK and CSSL8-95 also had lower EL than KDML105 (Figure 4A). This finding illustrated that salt stress causes gravely affected membrane damage in KDML105 than PK and both CSSL8 lines. Similarly, a significant increase in MDA was found in KDML105 under salt stress treatment whereas no significant change was noted in the case of PK and both CSSL8 lines (Figure 4B). These results indicated that less membrane damage was found in PK and both CSSL8 lines. In the study of salt stress response in four rice genotypes, salt-tolerant (PK and Rashpanjor) and salt sensitive (FR13A and IR42), the results showed that less amount of MDA was accumulated in PK and Rashpanjor compared to FR13A and IR42 when plants were treated with NaCl (Sarkar et al., 2013). Based on these two oxidative damage indicators, the two CSSLs are assumed to be less affected by salt stress than their genetic background genotype, KDML105.

### CSSL candidate genes associated with salt-tolerance and their co-expressed genes

To investigate possible mechanisms and pathways that might link to and promote salt tolerance ability in the CSSL8 lines within the genomic regions RM447 and RM3480, we constructed co-expression networks to aid visualization and cluster the putative genes with their co-expressed genes, based on their expression pattern similarity, and assigned functional categories using GO annotation. Compared to Arabidopsis, the GO annotation of rice is generally known to be not as well-characterized and sparse. In addition, smaller sets of available expression and transcriptomic data than those of Arabidopsis also mean the co-expression network analyses in rice might not be able to identify the genes involving in stress responses with the same accuracy. However, these bioinformatic analyses would at least suggest possible mechanisms and pathways that might be involved in salt stress regulation in these CSSLs, which could be investigated further.

#### Candidate genes associated with salt-tolerance in RM447

Co-expression network of RM447 reveals biological functions and mechanisms that are enriched in the genes showing similar expression patterns to the putative genes identified in the RM447 regions, which in turn might suggest possible functions of these putative genes as well. Interesting putative genes include Os08g41990, which encodes aminotransferase, located in chloroplast, shown to be involved in chlorophyll biosynthetic process (*P* = 0.0016), and also functions in tetrapyrrole biosynthetic process (*P* = 0.0006, GO term enrichment) and porphyrin biosynthetic process (*P* = 0.0012) (Cao et al., 2012). This gene shows 100% similarity to glutamate-1-semialdehyde 2,1-aminomutase 2 in *Arabidopsis* (Kawahara et al., 2013). This enzyme is also associated with tetrapyrrole synthesis in plants (Grimm, 1990). It has been reported that tetrapyrrole biosynthesis, especially the heme branch of the pathway, plays a crucial role in mediating intracellular drought stress signaling and activating ROS scavenging system under drought stress (Nagahatenna et al., 2015). Moreover, Os08g41990 is positively co-expressed with several genes under abiotic stress conditions. The genes co-expressed with Os08g41990 were identified as Clp proteases that are important for chloroplast function and plant development (Clarke, 2012). Another protease group identified in co-expression network was Deg protease. Similar to Clp protease, in rice, it was reported that Deg protease protein is not only necessary for chloroplast development but also necessary for photosystem II repairing when plants are grown under high temperatures condition (Zheng et al., 2016). The data obtained in this work showed that chlorophyll content in salt-stressed CSSLs did not decline significantly compared to untreated plants (Figure 3F). This may be because a set of chlorophyll biosynthetic related-genes were not repressed by salt stress. In our previous report, the salt-tolerant CSSL line harboring DT-QTL and the DT-QTL donor, showed less decrease in chlorophyll content compared to salt susceptible CSSL line (Nounjan et al., 2016).

Referring to the co-expression network of RM447 (Figure 7), another interesting candidate gene is Os08g42400, together with its 45 co-expressed genes. This gene encoded no apical meristem protein in rice might act as NAC protein (Kawahara et al., 2013). The gene Os08g42400 might have a regulatory function involved in transcriptional regulation of other downstream abiotic stress-responsive genes including salt-responsive genes. It is generally well-known that NAC transcription factors stimulate abiotic stress tolerance in plants. The study by Hong et al. (2016) presented that stress-responsive NAC transcription factor overexpressed in rice resulted in increased drought and salt tolerance.

#### Candidate genes associated with salt-tolerance in RM3480

For RM3480, our co-expression network analysis suggested that Os08g43230 and Os08g43440 might be novel candidate genes promoting abiotic stress tolerance. Although Os08g43230 has not been annotated in rice yet, according to the Arabidopsis Information Resource (TAIR) database, this gene might be located in chloroplast (TAIR, www.arabidopsis.org). However, the roles of those genes under abiotic stress need to be further investigated for increasing the understanding of it functions. For Os08g43440, it encodes cytochrome P450, was predicted to be involved in oxidation reduction (*P* = 0.0692) (Cao et al., 2012). This gene shows 85% similarity to flavonoid 3-monooxygenase in *Zea mays* (Kawahara et al., 2013). Cytochrome P450 plays many roles in plant development, biotic and abiotic defense system (Jun et al., 2015). It has been reported that cytochrome P450 monooxygenase involved in salt tolerance in *Arabidopsis* (Mao et al., 2013). This evidence suggested that Os08g43440 found in our co-expression network may be associated to salt stress tolerance in CSSL rice.

### Possible mechanisms associated with salt tolerance ability in CSSL rice

Phenotypic observations are controlled by genetic region segregation. Stress-related phenotypes of crop plants are influenced by specific QTL (Liseron-Monfils et al., 2015). Combination of physiological response to salt stress and application of computational method can be used to identify the specific biological processes associated with salt tolerance in CSSL rice.

#### Protecting ROS formation

According to the co-expression network, genes involved in iron (Fe)-sulfur (S) cluster assembly (Os03g20010, Os06g47940, and Os06g05400) were found to have correlation expression with genes in two DT-QTL segments (Os08g42400, Os08g43440, and Os08g43560) (Supplementary D). In plants, Fe-S cluster proteins are involved in the processes of photosynthesis, nitrogen and sulfur assimilation, electron transfer, and regulation of gene expression (Abdel-Ghany et al., 2005). Iron-sulfur (Fe–S) proteins are found in the plastids, mitochondria, cytosol, and nucleus, where they are essential for numerous physiological and developmental processes (Balk and Pilon, 2011). Wallace et al. (2004) suggested that ROS could damage Fe–S clusters protein. When the assembly of Fe–S clusters was released (by ROS), free iron was able to catalyze the production of more ROS in the Haber–Weiss cycle (Halliwell and Gutteridge, 1984; Balk and Pilon, 2011). It is known that plants can generate ROS under salt stress. Lots of experiments indicated that salt-tolerant cultivars (PK) had less content of H_2_O_2_ compared to salt-sensitive cultivars (Nounjan and Theerakulpisut, 2012; Sarkar et al., 2013). This might be due to better protection of Fe–S proteins in salt-tolerant cultivars. In this present study, EL and MDA were used as indicators for cellular damaging by ROS. There is less increase in MDA content in CSSL8-94 and CSSL8-95 (including PK) as compared to KDML105 under salt stress (Figure 5B). In accordance with those evidences, it has been proposed that Fe–S proteins may play a crucial role in protecting CSSL8 lines under salt stress. Lower accumulation of MDA under salt stress condition was also observed in salt-tolerant CSSL line compared to salt sensitive CSSL line (Nounjan et al., 2016).

#### Sugar accumulation for osmotic adjustment

Several gene functions in sugar synthesis process were observed in two co-expression networks such as gene involved in inositol-1-monophosphatase (Os02g07350), fructose 2,6-bisphosphate metabolic process (Os01g13570), malate metabolic process (Os03g56280, Os08g44810) and UDP-glucose metabolic process (Os03g25960). Expression of these genes is linked with Os08g41990, Os08g43230, and Os08g43440 that are genes located in both DT-QTLs. When plants encounter several abiotic stresses (drought, salinity, heat, and chilling) they usually accumulated high level of compatible solutes needed for osmotic adjustment. The significant increase in sugar content, as observed in this study (Figure 4D), might be due to the action of genes in sugar biosynthesis process in both CSSL8 lines under salt treatment.

## Conclusion

Physiological observation of both CSSLs for salt stress tolerance by consideration of growth, Na^+^/K^+^ ratio, water status, osmotic adjustment, photosynthetic parameters, EL, MDA, proline and sugar accumulations could suggest that CSSL8-94 and CSSL8-95 showed better salt-tolerant than their recurrent parent, KDML105. Salt tolerance mechanisms in CSSL8-94 and CSSL8-95 are associated to better protection of photosynthesis systems, osmotic adjustment (via accumulation of compatible solutes) and more efficient protection of membrane damage. This finding indicated that both CSSL lines which are tolerant to both drought and salt stress have a good potential to be used to breed more improved lines of KDML105 by gene pyramiding in the future.

Possible mechanism and identification of candidate genes involved in salt tolerance in DT-QTL segments were explored using co-expression networks. The results suggested that Os08g419090, a gene involved in tetrapyrrole and porphyrin biosynthetic process (chlorophyll biosynthetic process), Os08g43230 and Os08g43440 (encoded TraB family protein and cytochrome P450, respectively) might play vital roles in salt stress tolerance. Therefore, it is interesting to study the functional analysis of those candidate genes in depth to find out more information about its functions in promoting salt and drought tolerance characteristic in rice.

## Author contributions

NN, VC, and PT conceived and designed the experiments. NN performed the experiments, data collection and analyzed the data. PC and VC analyzed the data. NN, VC, and PT wrote manuscript. JS, TT, and SC interpretation of results and critically revised the manuscript. All authors read and approved the manuscript.

### Conflict of interest statement

The authors declare that the research was conducted in the absence of any commercial or financial relationships that could be construed as a potential conflict of interest.
