# Supplementary material for: High Performance of Photosynthesis and Osmotic Adjustment Are Associated With Salt Tolerance Ability in Rice Carrying Drought Tolerance QTL: Physiological and Co-expression Network Analysis
Source: Front Plant Sci. 2018 Aug 6;9:1135. doi: 10.3389/fpls.2018.01135 (PMC6088249; doi:10.3389/fpls.2018.01135)
Supplement: Supplementary file 7 [file Data_Sheet_1.PDF]

Supplementary G: Comparison of significant GO terms between results from ROAD and AgriGO for DT-QTL RM447 and 79 random genes

GO enrichment tool: AgriGO
Background/Reference: Rice TIGR gene model
Species: *Oryza sativa* Japonica
GO type: Completed GO
GO category: Biological Process

Figure S1. Comparison of significant GO terms between results from ROAD and AgriGO for DT-QTL RM447

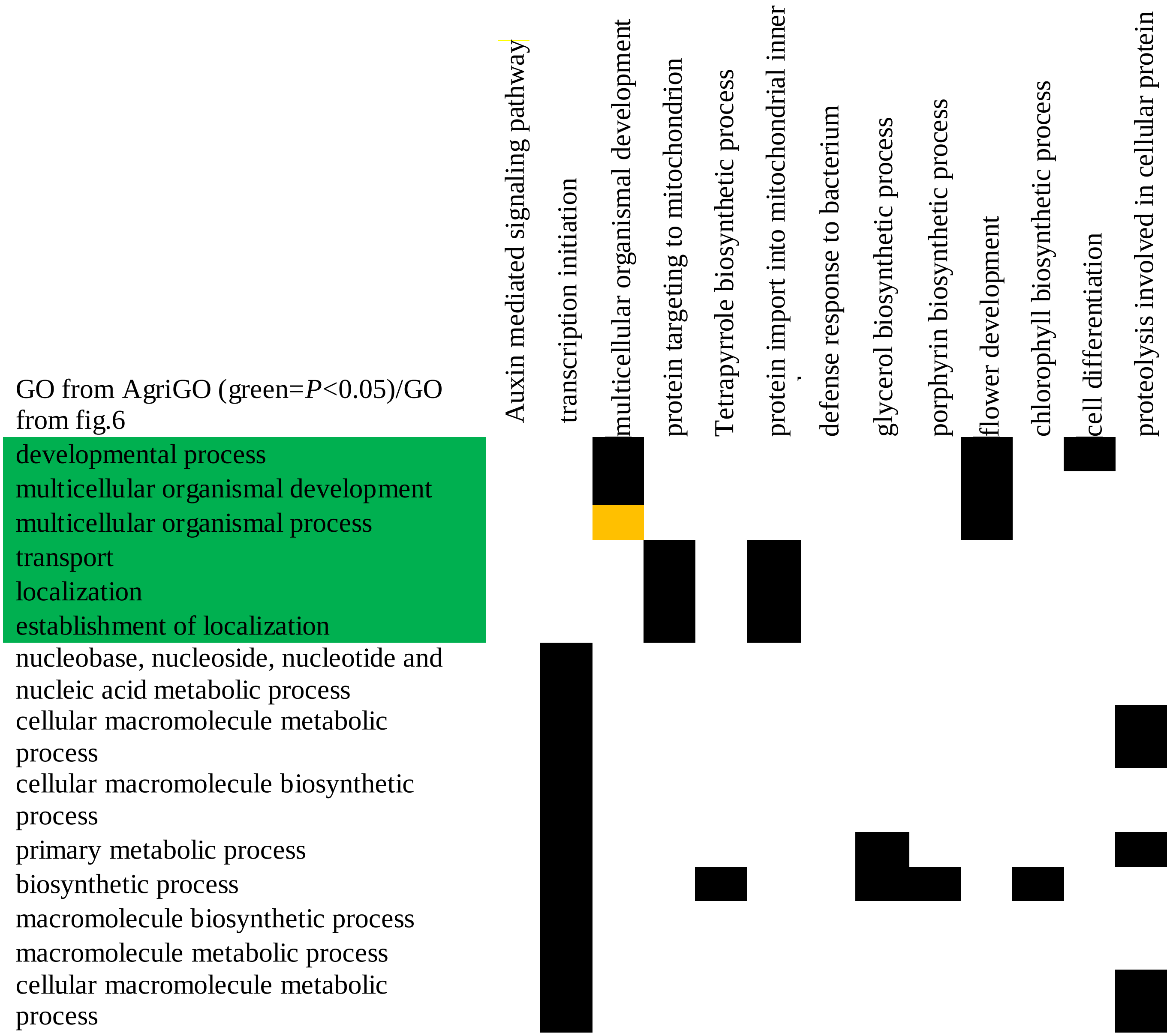

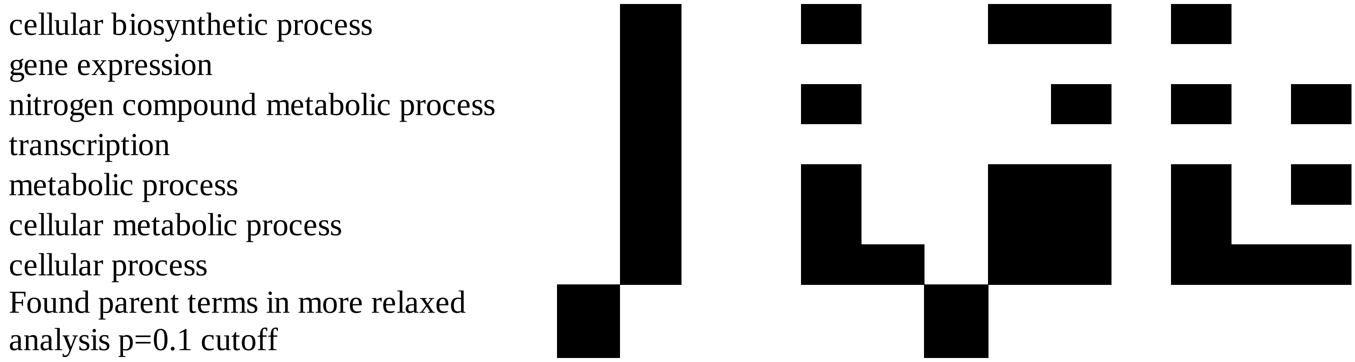

Figure S2. Comparison of significant GO terms between results from ROAD and AgriGO for DT-79 random genes

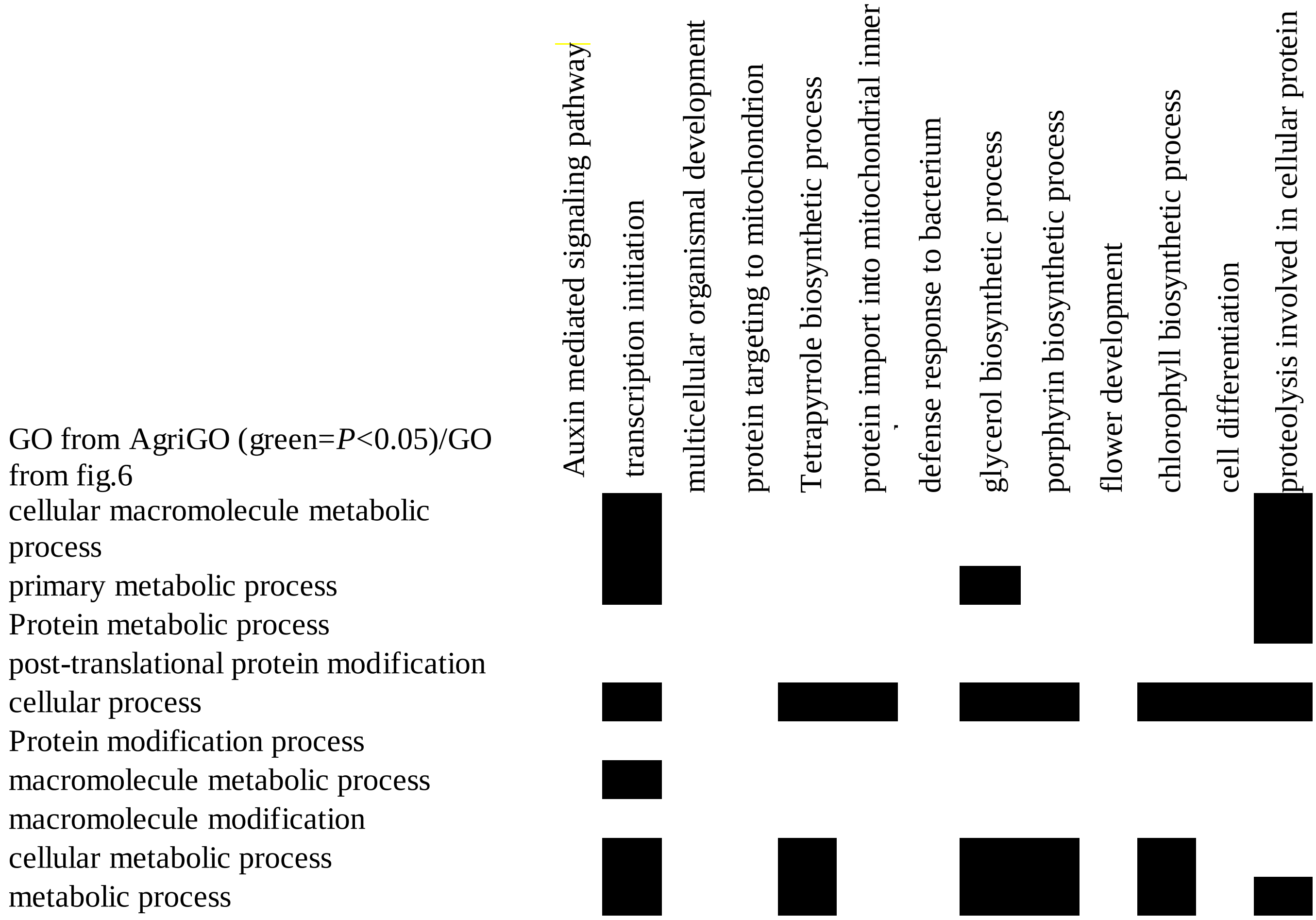

Figure S1 and S2 Parent-child GO terms association. In each table, the row shows significant/non-significant GO terms from AgriGO (green rows are significant terms with p-value<0.05), and significant GO terms from ROAD are in column. Color in each grid indicates a relationship between the terms in its respective row and column; **black**: a pair with parent-child relationship, **yellow**: a pair with same term.

Interpretation

According to the two tables presented here, there are differences between RM447 enrichment and 79 randomly picked genes.

1) RM447 has 6 significant terms while the random gene set has none. This emphasizes the unique function of this region.

2) Even though some of the non-significant terms from RM447 and random genes overlap, there are also several different members. For example, RM447 has synthetic and gene expression related terms while randomly picked group has protein modification related terms, which have no association terms from ROAD result (e.g. Auxin mediated signaling pathway, multicellular organismal development, protein targeting to mitochondrion, defense response to bacterium, and flower development).
