# Supplementary material for: High Performance of Photosynthesis and Osmotic Adjustment Are Associated With Salt Tolerance Ability in Rice Carrying Drought Tolerance QTL: Physiological and Co-expression Network Analysis
Source: Front Plant Sci. 2018 Aug 6;9:1135. doi: 10.3389/fpls.2018.01135 (PMC6088249; doi:10.3389/fpls.2018.01135)
Supplement: Supplementary file 8 [file Data_Sheet_2.PDF]

Supplementary H: Comparison of significant GO terms between results from ROAD and AgriGO for DT-QTL RM3480 and 78 random genes

GOenrichment tool: AgriGO
Background/Reference: Rice TIGR gene model
Species: *Oryza sativa* Japonica
GO type: Completed GO
GO category: Biological Process

Figure S1. Comparison of significant GO terms between results from ROAD and AgriGO for DT-QTL RM3480

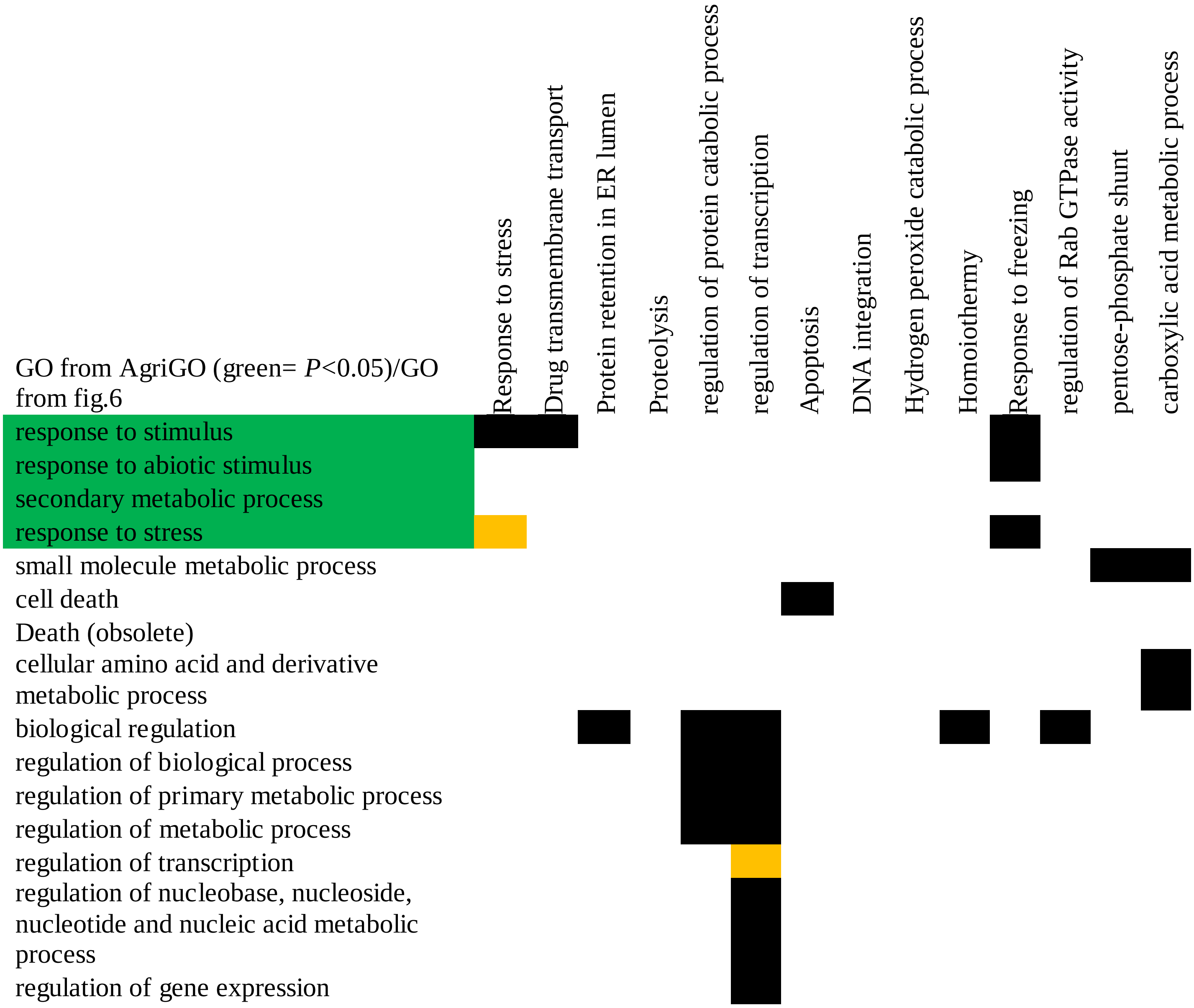

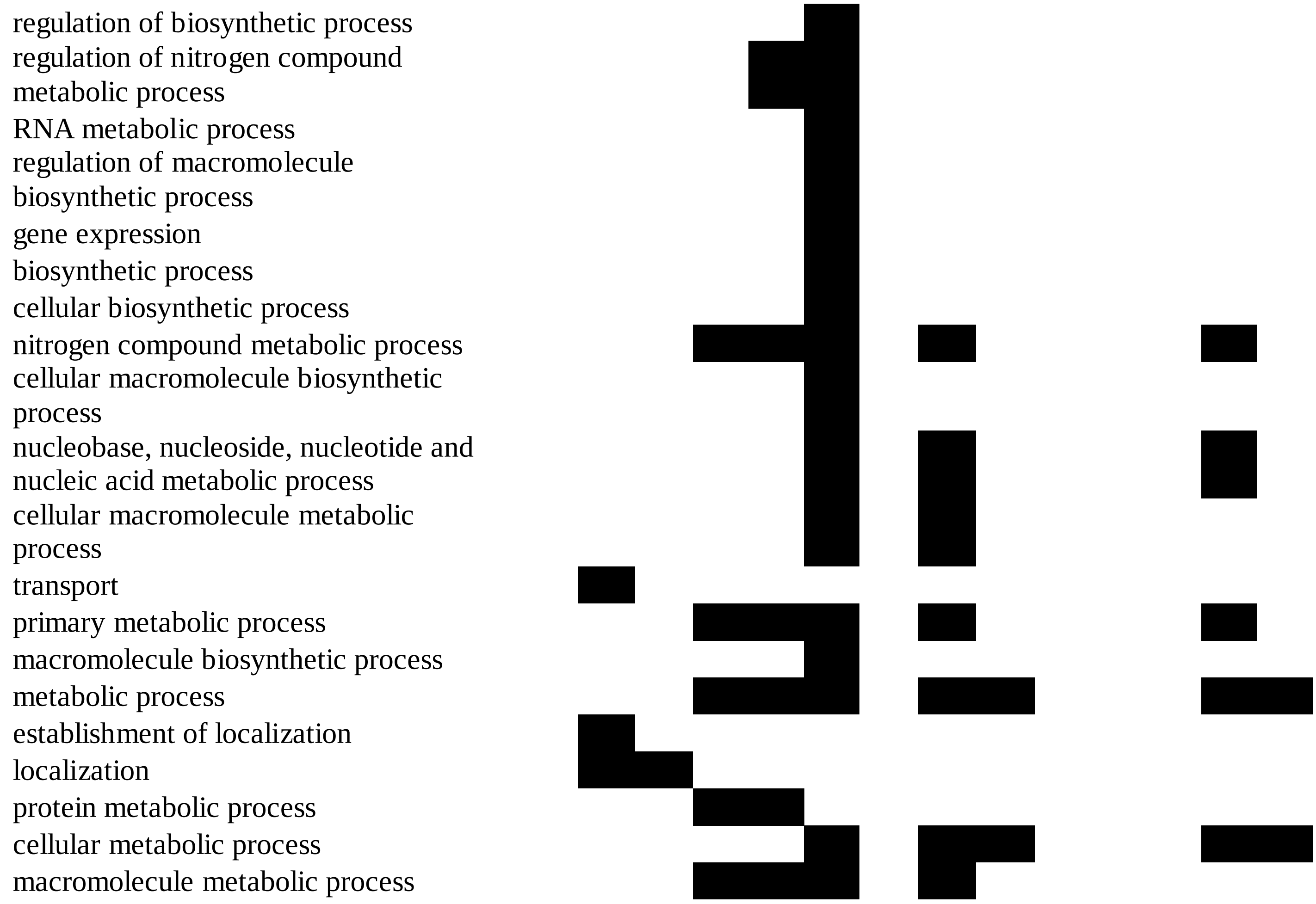

Figure S2. Comparison of significant GO terms between results from ROAD and AgriGO for DT-78 random genes

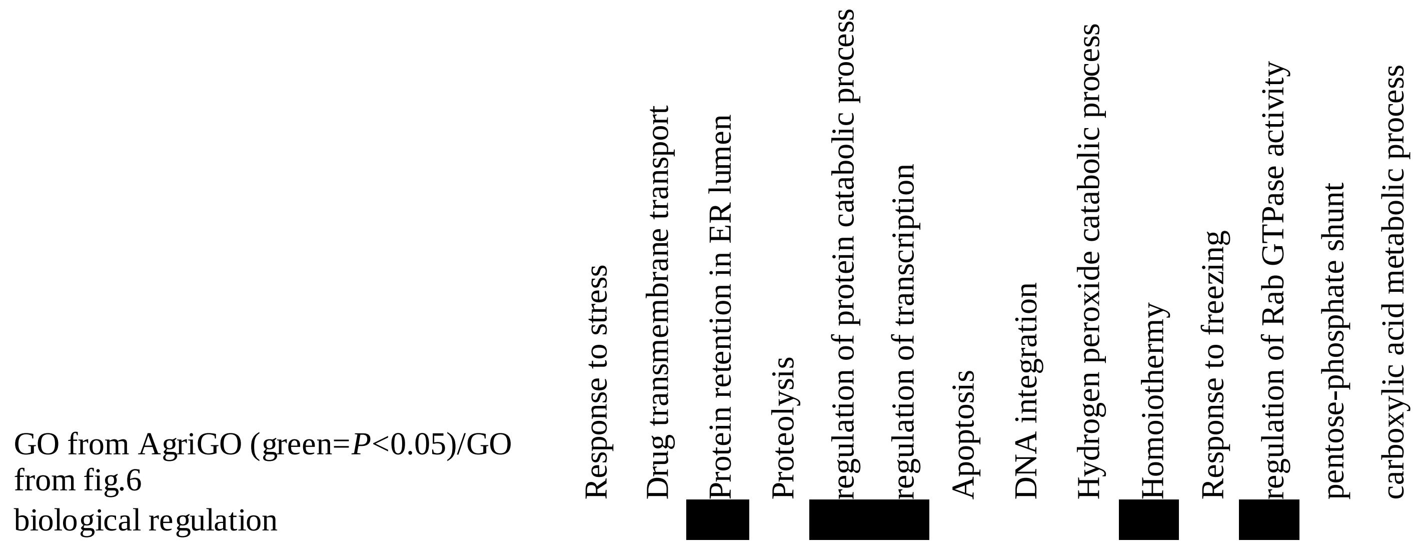

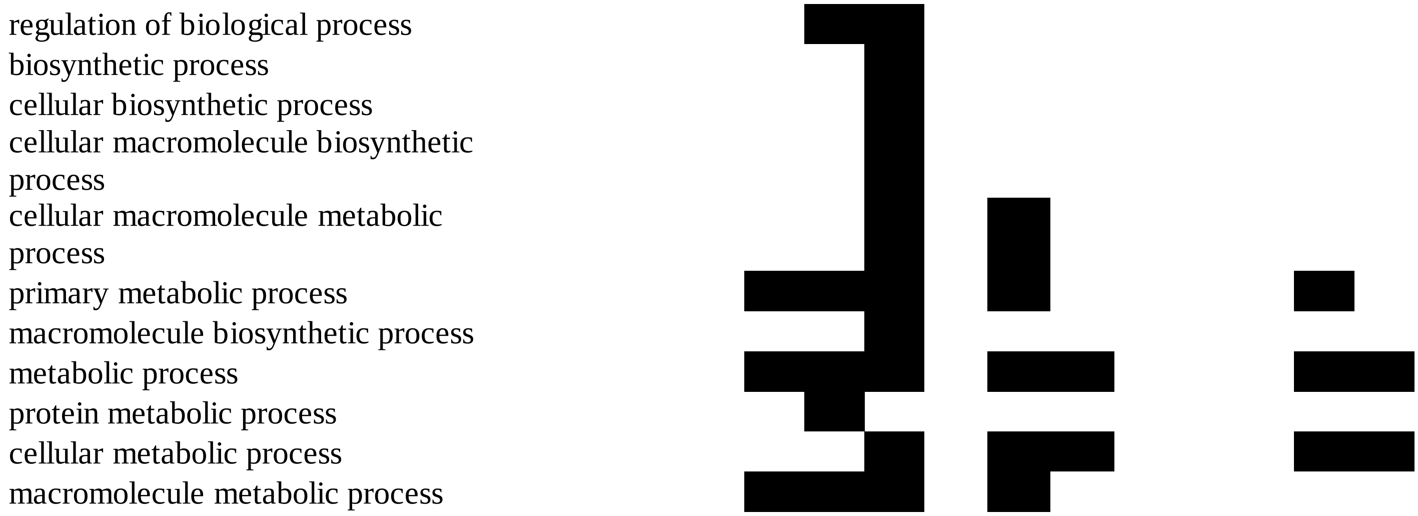

Figure S1 and S2 Parent-child GO terms association. In each table, the row shows significant/non-significant GO terms from AgriGO (green rows are significant terms with p-value<0.05), and significant GO terms from ROAD are in column. Color in each grid indicates a relationship between the terms in its respective row and column; **black**: a pair with parent-child relationship, **yellow**: a pair with same term.

Interpretation

According to the two tables presented here, there are some differences between the GO enrichment of the RM 3480 genes and 78 randomly picked genes.

1) RM3480 has 4 significant terms while the random gene set has none. This emphasizes the unique function related to stress responses of this region.

2) RM3480 has regulation of biosynthetic, gene expression related terms, cell death, small-molecule metabolic process, cellular amino acid and derivative metabolic process, and localization, which are not presented in the result from random gene set.

3) The results from randomly picked 78 genes could not find any term to associate with some GO terms from ROAD results such as Response to stress, Drug transmembrane transport, Apoptosis, and Response to freezing.
