# Supplementary figures and images for: High Performance of Photosynthesis and Osmotic Adjustment Are Associated With Salt Tolerance Ability in Rice Carrying Drought Tolerance QTL: Physiological and Co-expression Network Analysis
Source: Front Plant Sci. 2018 Aug 6;9:1135. doi: 10.3389/fpls.2018.01135 (PMC6088249; doi:10.3389/fpls.2018.01135)

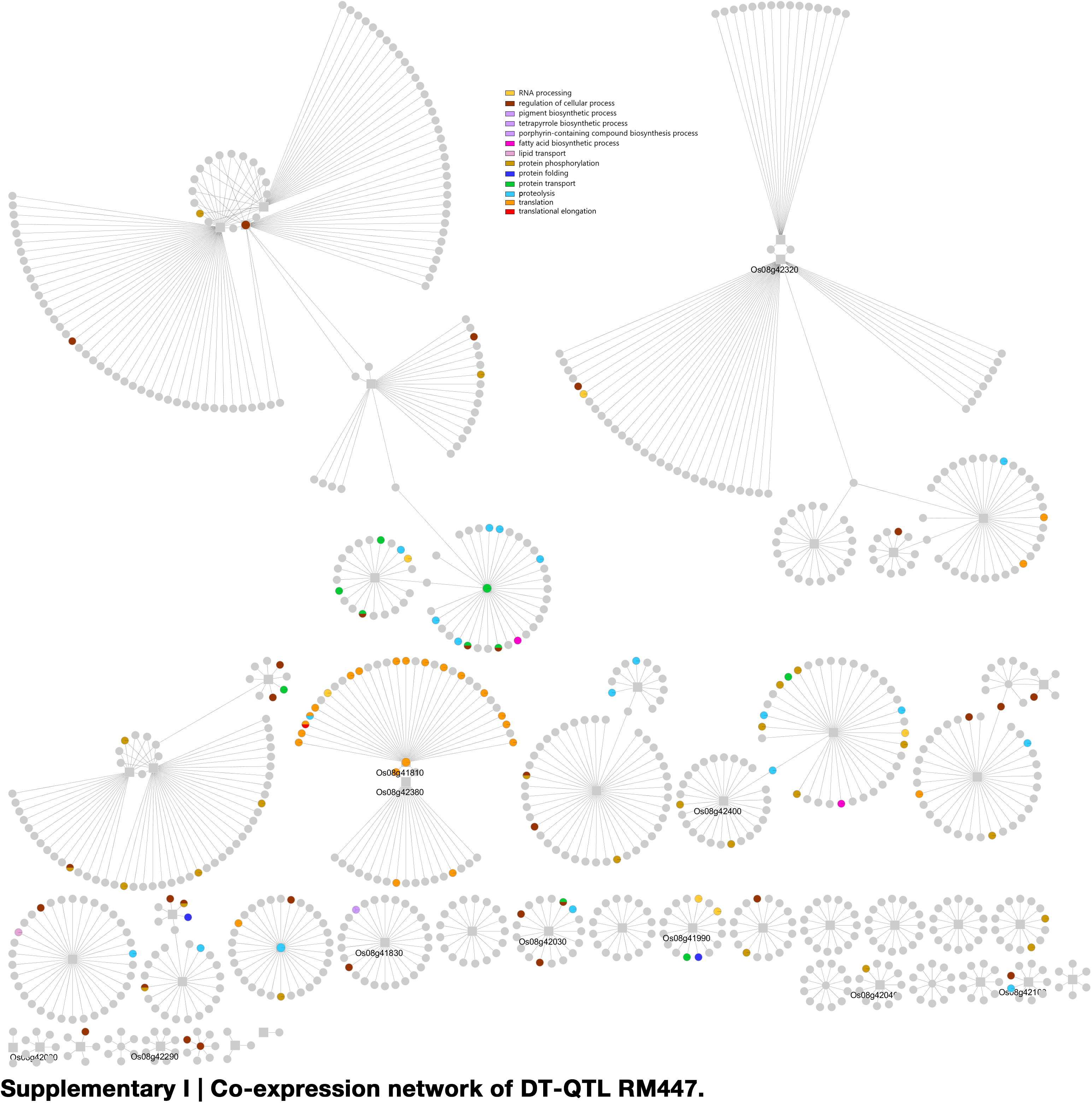

Supplement: Supplementary file 9 [file Image_1.TIF]

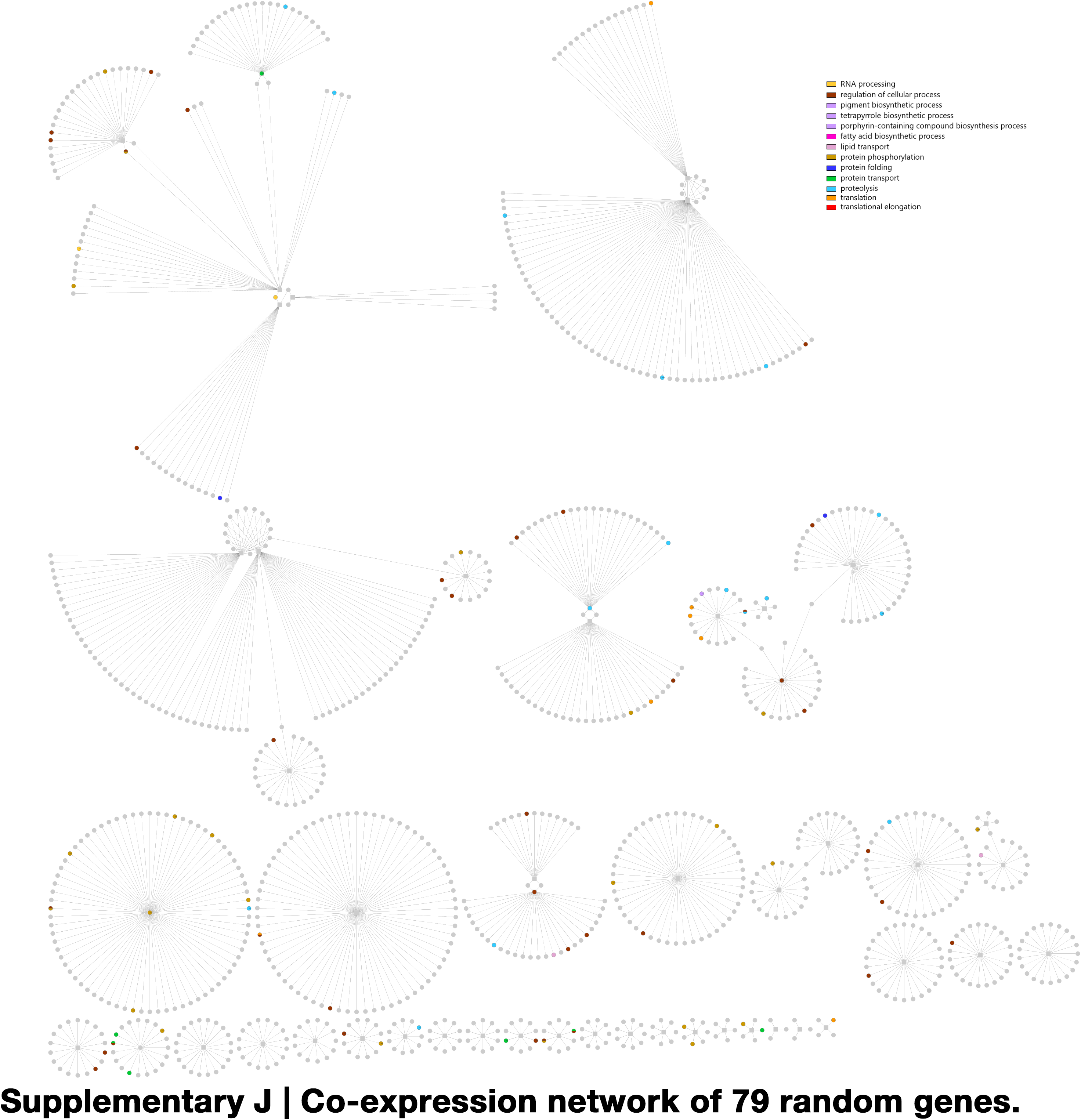

Supplement: Supplementary file 10 [file Image_2.TIF]

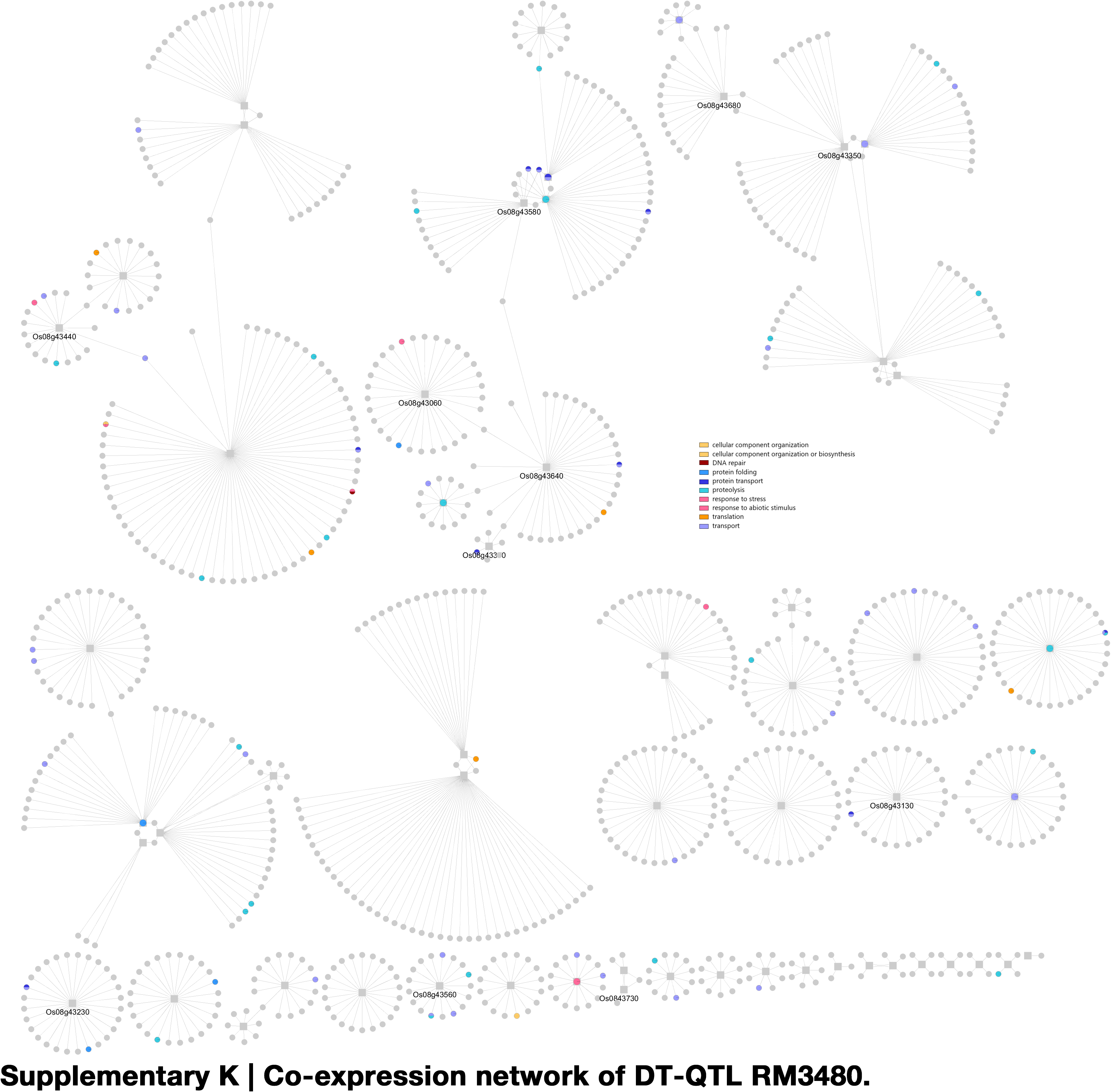

Supplement: Supplementary file 11 [file Image_3.TIFF]

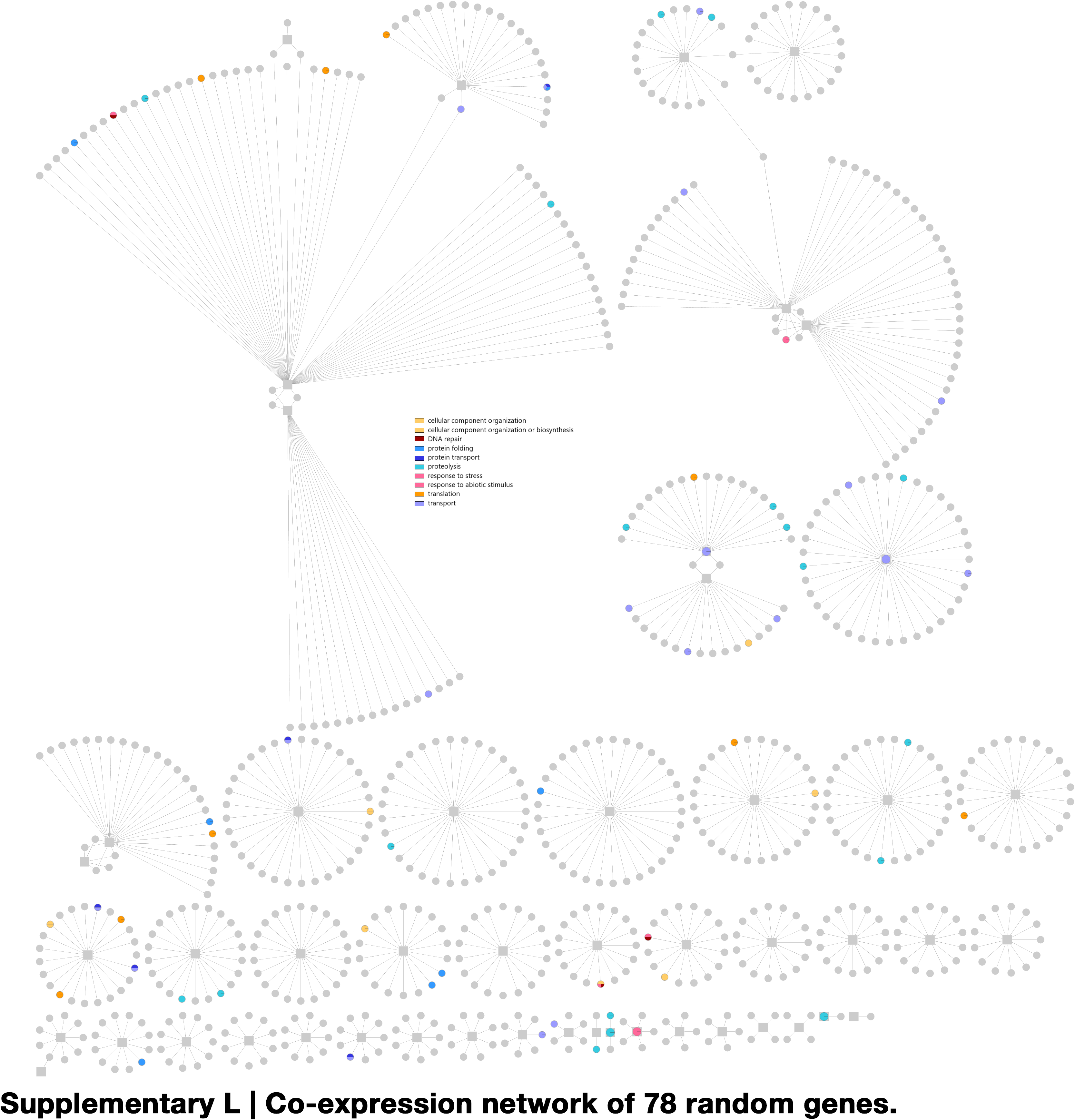

Supplement: Supplementary file 12 [file Image_4.TIF]

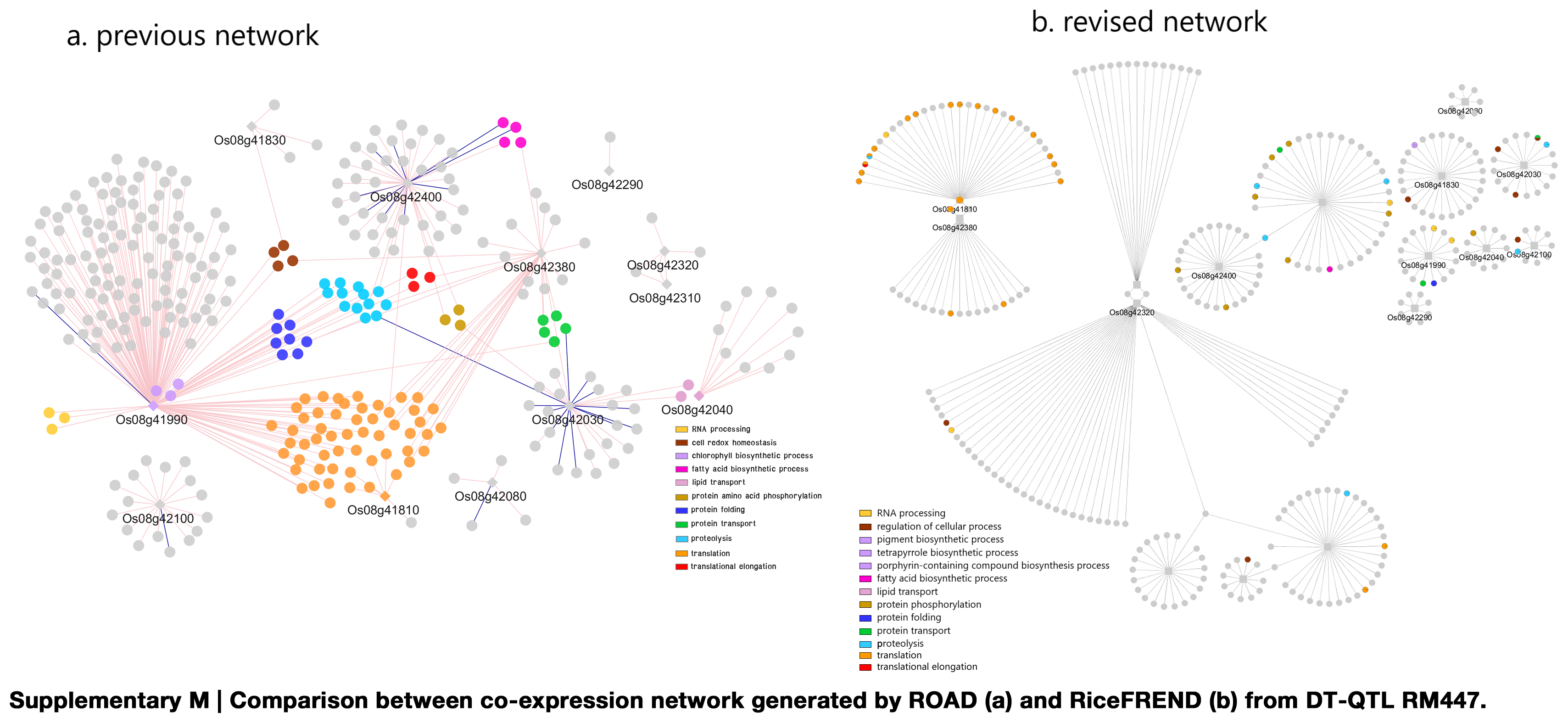

Supplement: Supplementary file 13 [file Image_5.TIF]

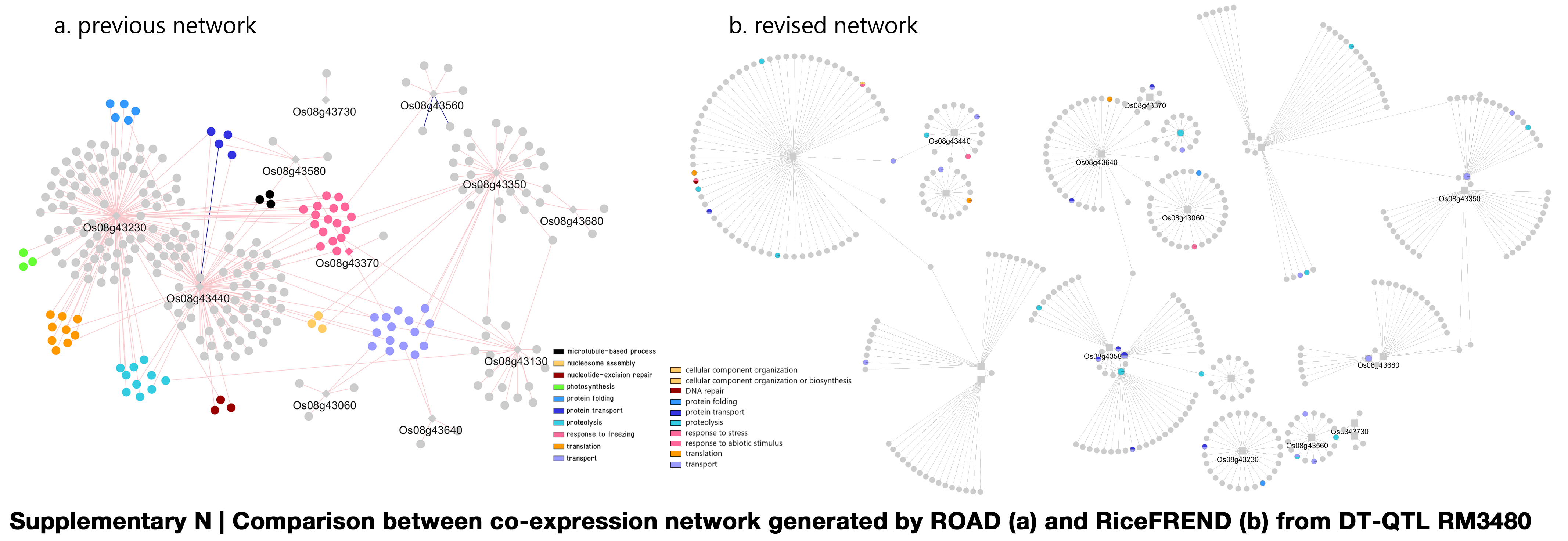

Supplement: Supplementary file 14 [file Image_6.TIF]

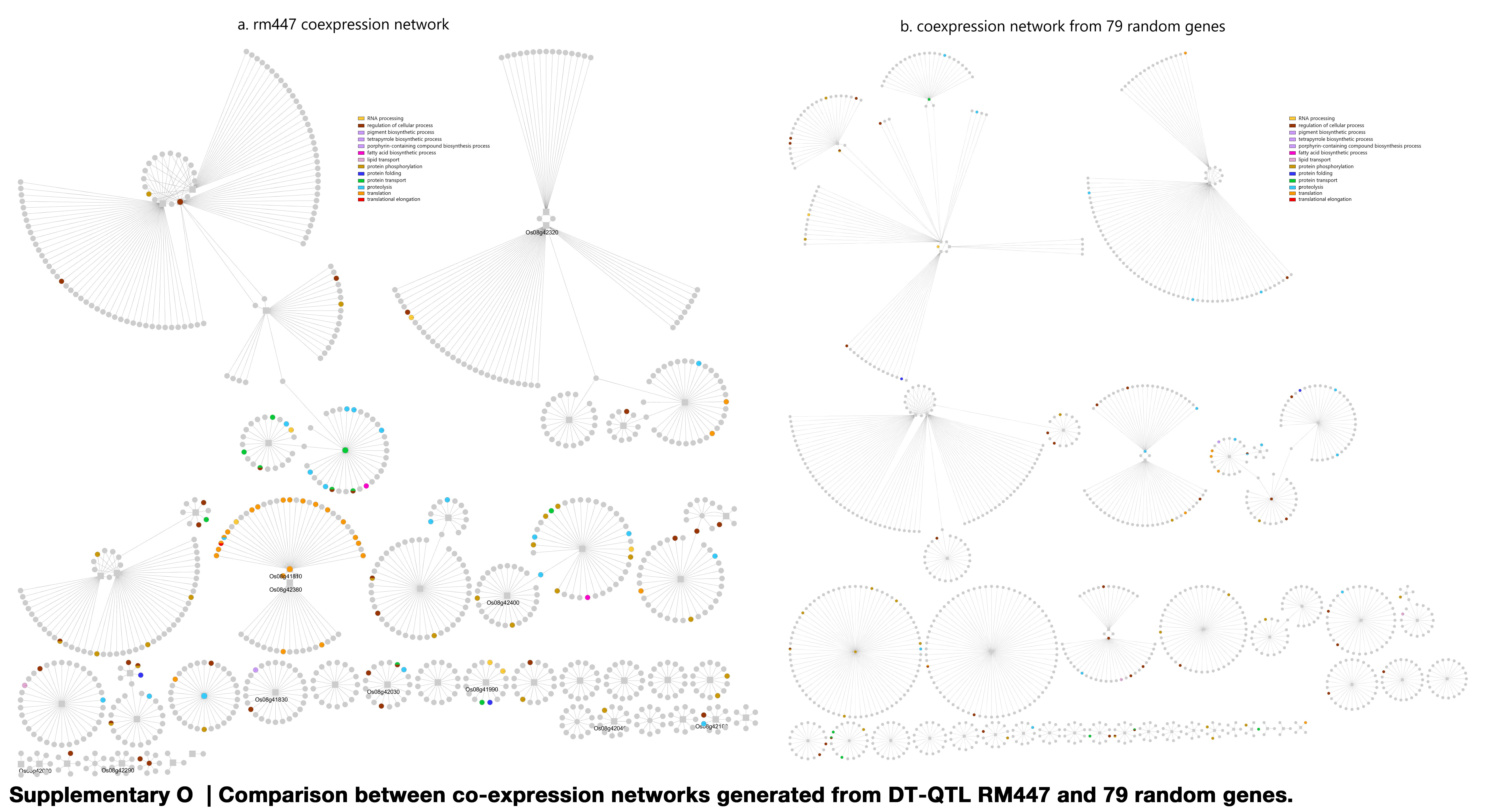

Supplement: Supplementary file 15 [file Image_7.TIF]

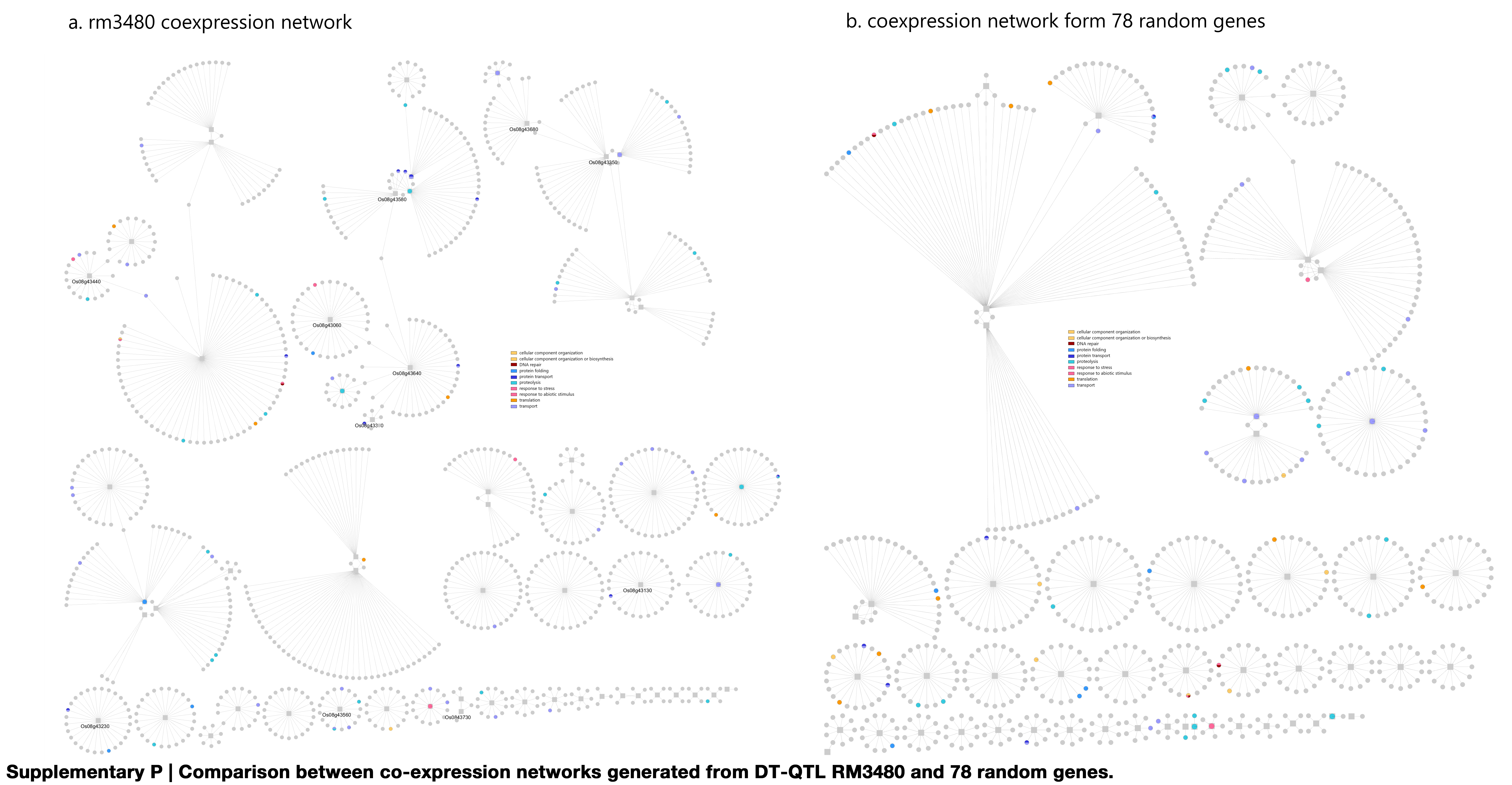

Supplement: Supplementary file 16 [file Image_8.TIF]
